# Parallelized Latent Dirichlet Allocation Provides a Novel Interpretability of Mutation Signatures in Cancer Genomes

**DOI:** 10.3390/genes11101127

**Published:** 2020-09-25

**Authors:** Taro Matsutani, Michiaki Hamada

**Affiliations:** 1 Graduate School of Advanced Science and Engineering, Waseda University, 55N-06-10, 3-4-1, Okubo Shinjuku-ku, Tokyo 169-8555, Japan; taromss@moegi.waseda.jp; 2 Computational Bio Big-Data Open Innovation Laboratory (CBBD-OIL), National Institute of Advanced Industrial Science and Technology (AIST), Tokyo 169-8555, Japan; 3 Japan Society for the Promotion of Science (JSPS), Tokyo 102-0083, Japan; 4 Graduate School of Medicine, Nippon Medical School, Tokyo 113-8602, Japan

**Keywords:** cancer genome, mutation signature, Bayes modeling, latent Dirichlet allocation

## Abstract

Mutation signatures are defined as the distribution of specific mutations such as activity of AID/APOBEC family proteins. Previous studies have reported numerous signatures, using matrix factorization methods for mutation catalogs. Different mutation signatures are active in different tumor types; hence, signature activity varies greatly among tumor types and becomes sparse. Because of this, many previous methods require dividing mutation catalogs for each tumor type. Here, we propose parallelized latent Dirichlet allocation (PLDA), a novel Bayesian model to simultaneously predict mutation signatures with all mutation catalogs. PLDA is an extended model of latent Dirichlet allocation (LDA), which is one of the methods used for signature prediction. It has parallelized hyperparameters of Dirichlet distributions for LDA, and they represent the sparsity of signature activities for each tumor type, thus facilitating simultaneous analyses. First, we conducted a simulation experiment to compare PLDA with previous methods (including SigProfiler and SignatureAnalyzer) using artificial data and confirmed that PLDA could predict signature structures as accurately as previous methods without searching for the optimal hyperparameters. Next, we applied PLDA to PCAWG (Pan-Cancer Analysis of Whole Genomes) mutation catalogs and obtained a signature set different from the one predicted by SigProfiler. Further, we have shown that the mutation spectrum represented by the predicted signature with PLDA provides a novel interpretability through post-analyses.

## 1. Introduction

Cancer is a major lifestyle disease, and the entire mechanism underlying carcinogenesis is unclear. Cancer genomes include numerous mutations caused by various mutational processes including smoking and exposure to ultraviolet radiation [1]. These mutational processes have their own unique mutational patterns. For example, UV radiation frequently causes cytosine-to-thymine substitutions [2]. Thus, a mutational distribution corresponding to one mutational process is called a mutation signature.

Elucidation of mutation signatures would provide insights into the mechanism underlying carcinogenesis [3]; however, the overall landscape of mutation signatures remains unclear.

**Figure 1:**
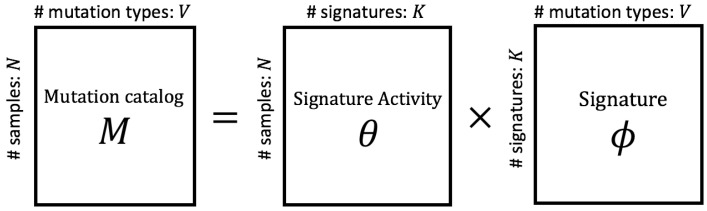
An illustration of matrix factorization of mutation catalogs to predict mutation signatures. As the observed, *M*, mutation catalogs of cancer patients are provided, and each element shows how many mutations are present in the genome in each sample. When extracting mutation signatures from mutation catalogs, it is assumed that mutations in each sample are represented by a combination of multiple signatures. Matrix *M* is approximated to the product of the signature activity matrix θ and signature distribution matrix ϕ. In non-negative matrix factorization-based methods, θ and ϕ are predicted to minimize the Frobenius norm of the matrix *M* and the product θϕ. We developed a new signature estimation method following this method.

To predict mutation signatures, Alexsandrov, et al. (2013) used non-negative matrix factorization (NMF) [4] to decompose the mutation catalog matrix [5,6] and revealed numerous mutation signatures [5,6,7,8,9,10]. They applied their method, called SigProfiler [11], to large data provided by the ICGC/TCGA Pan-Cancer Analysis of Whole Genomes (PCAWG) Network [12], and their predicted signatures are reported in the COSMIC database (https://cancer.sanger.ac.uk/cosmic/signatures) and are available to everyone. Figure 1 shows the simple image of matrix factorization to predict mutation signatures. Based on these data, *M*, mutation catalogs of cancer patients have been obtained and represented by the *N* (the number of samples) × *V* (the number of mutation types) matrix. Each element of this matrix, $m_{nv}$, shows how many *v*th mutations (1≤v≤V,v∈N) are present in the genome of the *n*th sample (1≤n≤N,n∈N). Several possibilities exist for selecting the type of mutations, such as the inclusion of indels (insertions and deletions) and genome reconstruction; however, we focused on only SNPs and their surrounding bases (e.g., A[T>C]G) in this study (V=96). Assuming that mutations in the cancer genome have accumulated through the effects of multiple mutation signatures, this matrix *M* can be interpreted as the product of θ, the matrix representing signature activities, and ϕ, the matrix representing mutational distributions for each signature. In NMF-based methods, θ and ϕ are predicted to minimize the Frobenius norm between the original mutation catalog *M* and the approximated product θϕ. For this formalized problem, numerous methods for signature prediction have been devised. In this matrix decomposition, it is necessary to regularize for likelihood and select the number of signatures (*K* in Figure 1) contributing to the composition of mutation catalogs because the degree of freedom of the model and the likelihood after parameter estimation continuously increases with an increase in *K*. To resolve this issue, EMu [13] and signeR [14] using BIC, which is one of the information criteria, and the method using latent Dirichlet allocation [15,16] have been developed, enabling prediction with greater mathematical stringency. Some mutation signatures are being confirmed through biological assays and are no longer simply concepts derived from in silico analyses, e.g., Zou et al. (2018) used CRISPR-Cas9 technology to knock out individual genes in vitro, and confirmed the existence of mutation signatures [17].

Although numerous methods are available for predicting signatures, many methods have one prominent limitation: mutation catalogs must be analyzed separately for each tumor type when decomposing catalogs to their combinations of signatures. Here, the “tumour type” is defined as the combination of the types of primary lesion and histology, which is denoted by (primary lesion)-(histology); for example, both “Skin-BCC” and “Skin-Melanoma” cancers occur in the skin as the primary lesion, but the tumor type is different because there is different histology between BCC (basal cell carcinoma) and melanoma. It is widely accepted that cancer genomes from different tumor types have different active signatures. Therefore, signature activity matrices become very sparse when the mutation catalog is not divided by each tumor type, which makes factorization difficult. Furthermore, this problem causes another problem. If we predict signatures using a mutation catalog for each tumor type, signatures with similar distributions among different tumor types are often obtained. In such cases, signature correspondence can be evaluated by clustering all the obtained signatures. Because numerous mutational processes involve multiple tumor types, signatures with similar mutational distributions are naturally derived from the same mutational process. However, it is important to determine which signature is the true distribution corresponding to that mutational process among the several predicted signatures. One possible solution to this problem is, for instance, to consider the average distribution for each signature as a true distribution; however, this is an ad hoc method.

To solve these problems, Alexandrov et al. have developed SigProfiler, which can predict signatures of multiple tumor types simultaneously [11]. This software uses “stability” (in other words. “reproducibility”) as an index to determine the number of mutation signatures in all mutation catalogs, and such modeling leads to more robust prediction against sparse activities. However, the threshold of stability is a hyperparameter that needs to be manually set, and the analysts must determine the appropriate value with heuristics. SignatureAnalyzer can also predict signatures with all mutation catalogs [18]. This is one of the extended models of NMF, and it decomposes a target matrix to minimize generalized β-divergence based on Bayesian theory [19]. As the objective function of this method includes a regularization term, the method can deal with a sparse matrix in a more natural form.

Here, we propose parallelized latent Dirichlet allocation (PLDA) model, a novel Bayesian model to simultaneously predict mutation signatures with all mutation catalogs. In this model, we prepare hyperparameters to directly model which signatures are probably active at which tumor type using labels like supervised topic model [20], and prevent the sparseness problems of signature activity.

## 2. Methods

### 2.1. Modeling Mutation Catalogs

The proposed method, PLDA, is an extended model of latent Dirichlet allocation (LDA) [21]. As indicated in the introduction, LDA has been used for signature modeling, and its flexible modeling using previous distributions is more preferable than other NMF-based methods. Furthermore, the number of signatures in the mutation catalog can be predicted by learning the parameters using the variational Bayes method [22].

**Figure 2:**
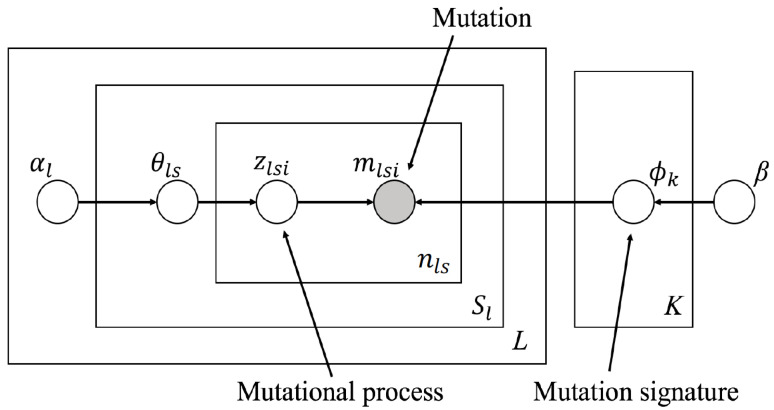
The graphical model of parallelized LDA. Parallelized LDA (PLDA) is an extended model of latent Dirichlet allocation. Compared with normal LDA, hyperparameter α is parallelized and shown as $\alpha_{l}$, which represents the bias of the types of signatures likely to appear at the *l*th tumor type. The signature activity of *l*th tumor type, $\theta_{ls}$, is generated from Dirichlet distribution with $\alpha_{l}$. Other parameters other than $\alpha_{l}$ represent the same object as the method using normal LDA [16].

Here, we set the hyperparameter vector of LDA for the number of tumor types, and attempted to represent the bias of the active signature for each tumor type. Figure 2 shows the graphical model of PLDA. A detailed notation of each parameter is provided in our previous study [16]. The vector $\theta_{ls}$ is the parameter of *K*-dimensional multinomial distribution, indicating the signature activity of the *s*th sample in *l*th tumour type ($1\leq s\leq S_{l},1\leq l\leq L,\;s\;\mathrm{and}\;l\in N$). Thereafter, $\theta_{ls}$ is generated from the Dirichlet distribution with hyperparameter vector $\alpha_{l}$, which is also a *K*-dimensional vector. Owing to the nature of the Dirichlet distribution, the expected activity of the *k*th signature is

(1) $$\begin{array}{c} E\left[\theta_{lsk}\right]=\frac{\alpha_{lk}}{\sum_{k^{\prime }=1}^{K}\alpha_{lk^{\prime }}} \end{array}$$

where α can be interpreted as a parameter representing the bias of signature activity. On modeling with normal LDA, α is a single vector (i.e., α is not $\left\{\alpha_{1},\alpha_{2},\cdots ,\alpha_{l},\cdots ,\alpha_{L}\right\}$); hence, such information regarding each sample (e.g., tumor type of sample) cannot be reflected. Since the types of active signatures vary among tumor types, it is difficult to simultaneously analyze all mutation catalogs. Therefore, we incorporated this bias into the model using different α values for each tumor type. Again, in Figure 2, *L* denotes the number of tumor types in all mutation catalogs, and each $\alpha_{l}$ is a *K*-dimensional vector showing the bias of the type of signature that probably appears at the *l*th tumor type.

In PLDA, model selection (determining the number of mutation signatures *K*) is possible by extending the variational Bayes method used in normal LDA. The variational Bayes method updates the parameters (θ,ϕ in Figure 2) to improve the objective function, variational lower bound, which is also utilized during model selection (cf. see [16]). The bottleneck part of the computational complexity with respect to time is the calculation of the responsibility from which signature each mutation is generated, $q(z_{lsi})$, and it follows $O(K\times \sum_{l=1}^{L}\sum_{s=1}^{S_{l}}n_{ls})$. If we independently consider the likelihood of each tumor type, variational lower bounds can be easily derived, and the number of signatures can be determined. Furthermore, hyperparameters (α,β in Figure 2) were obtained using the fixed point iteration method. The procedure to predict PLDA parameters is detailed in the Supplementary material. All source codes used in the following experiments are available in the Github repository (https://github.com/qkirikigaku/Parallelized_LDA).

### 2.2. The Property of PLDA

The advantage of predicting mutation signatures using our methods is that all signature sets can be obtained without post-processing such as clustering or merging after decomposition of mutation catalogs like SigProfiler and SignatureAnalyzer. All signatures can be simultaneously predicted using PLDA, and only one signature corresponding to one mutational process is obtained; hence, post-processing is not necessary. In addition, our method does not require the hyperparameter setting that was necessary in SigProfiler, which allows analysts to predict signatures with their own catalogs by easier operations.

Similarly, PLDA helps easily analyze signature activity across tumor types. If signatures are predicted for each tumor type with previously reported methods, the number of signatures (*K*) is determined by each tumor type, such that the activity dimension differs between them. However, with the proposed method, all signatures are simultaneously predicted, and the dimensions of signature activity are identical *K*. Consequently, we can directly apply dimensionality reduction methods including principal component analysis (PCA) and *t*-distributed stochastic neighbor embedding (*t*-SNE) [23] to activities among different tumor types, which may lead to the discovery of the relationships between tumor types.

Furthermore, more consistent analysis would be possible for tumor types with few samples. In general, factorization methods need many samples to learn parameters; hence, the previous method could not sufficiently analyze tumor types with only a few samples. Because the PLDA shares the same signature matrix ϕ among tumor types, it may reveal minor signatures specific to tumor types with few samples.

### 2.3. Comparison with Other Similar Models

Owing to its versatility, there exist many LDA-based models that are widely used in the field of bioinformatics [24,25]. Among them, supervised LDA [20] should be noted as an extended model closely related to this research. In supervised LDA, auxiliary information (i.e., metadata) of the document can be predicted based on the topic responsibility ($z_{s}$ in Supplementary Figure S1), thereby enabling parameter estimation in consideration of the auxiliary information. In the context of signature prediction, the auxiliary information is tumor types for each sample. However, in the original study [20], the auxiliary information is obtained from normal distribution, and hence the metadata used is limited to continuous values. Therefore, the direct application of this model to the problem focused in this study would be difficult. Moreover, if we adopt supervised LDA for our problem, the causal relationship that signature activity is determined by the tumor types is reversed. In supervised LDA, we update the model parameters in the learning process while treating the tumor types as unknown variables, and this is unreasonable because the tumor types are fixed and known information. In contrast, PLDA enables more intuitive modeling by preparing hyperparameters for each categorical auxiliary information. Thus, PLDA could be applicable in various cases of natural language processing as well as signature prediction, when the data contain categorical auxiliary information that determines topic distributions. See Supplementary Materials for the more detailed comparison between PLDA and supervised LDA.

## 3. Result

### 3.1. Simulation with Artificial Data Following Generation Process of PLDA

To verify the performance of PLDA, we conducted a simulation experiment using artificial data and compared it with the results obtained by previous methods (normal LDA, SigProfiler, SignatureAnalyzer). Artificial data are generated in accordance with the PLDA generation process with hyperparameters (α), as shown in Table 1. Herein, the value of hyperparameters α was set to reflect the actual feature that different signatures are active in different tumor types, and other parameters, $n_{ls}$ (the number of mutations for each sample), $S_{l}$ (the number of samples derived from each tumor type), and *L* (the number of virtual tumor types) were set to 1000, 100, and 5, respectively. Using the previous methods, a mutation catalog integrating datasets generated for each virtual tumour type is used (1st∼5th). Furthermore, five signatures were selected from COSMIC v3.1 (SBS1, SBS2, SBS3, SBS4, SBS5) (https://cancer.sanger.ac.uk/cosmic/signatures/SBS/index.tt) and used as mutational distributions ϕ to generate a data set similar to the actual mutation catalogs. We generated 100 data sets with the same setting according to Table 1, and compared the ability of PLDA and previous methods to extract signatures. See Supplementary Section S3 for more details on how to generate artificial mutation catalogs. For the previous methods SigProfiler (https://github.com/AlexandrovLab/SigProfilerExtractor) and SignatureAnalyzer (https://github.com/broadinstitute/getzlab-SignatureAnalyzer), we used publicly available implementation and adopted default values for the hyperparameters. In addition, simulation experiments for all the methods were repeated 10 times to avoid local minima, and the best results are shown in this paper.

Regarding model selection (the ability to determine the number of signatures), since artificial data sets use five signatures, K=5 would be predicted. Table 2 shows the results of model selection with PLDA and previous methods. Here, this table shows how many times each the number of signatures was selected ($K_{\mathrm{predicted}}$) of a total of 100 times. From this table, we can see that $K_{\mathrm{predicted}}=5$ (the correct value) could be obtained many times in the order of PLDA = SignatureAnalyzer, SigProfiler, normal LDA. In particular, PLDA and SignatureAnalyzer were able to predict the correct value for all datasets, and normal LDA could never predict the correct value. We think that SignatureAnalyzer performs better because its objective function contains a regularization term and it is more robust to sparse data than other existing methods.

Next, we examined whether each method could predict correct mutational distributions. Table 3 shows the results of matching the predicted signatures to correct signatures based on the cosine distance for all the datasets. The upper table shows the number of times each correct signature was predicted (threshold of cosine distance is 0.1), and the lower table shows its summary. In the lower table, the “Duplicated” column shows the number of predicted signatures that matched the corresponding correct signatures; however, all the methods could avoid such a solution. The “Not Matched” column also shows the number of predicted signatures which did not match any of the correct signatures, and these columns indicate the number of false positives. Ideally, all signatures are predicted 100 times, and “Duplicated” and “Not Matched” are expected to be zero. In 100 data sets, PLDA and SignatureAnalyzer predicted the correct signature with one-to-one correspondence without any errors.

Furthermore, we observed that PLDA outperforms SigProfiler and SignatureAnalyzer in certain cases where it is difficult to predict signatures; Table 4 shows an example of one such case. In this experiment, the number of signatures was K=10, and all of them were COSMIC ver3.1 signatures with small indices. In a scenario, where there are only two samples from 6th to 10th artificial tumor types ($S_{l}=2,6\leq l\leq 10$), and other settings are the same as in the previous example, we generated 100 data sets as described in the previous example and applied PLDA, SignatureAnalyzer, SigProfiler, and LDA. In model selection, all methods could not stably predict the correct number of signatures (K=10) as shown in Table 5. When we matched the predicted signatures with the correct signatures like in the previous setting, we confirmed that PLDA outperforms the existing methods as shown in Table 6. It is notable that with PLDA, the number of “Correct Matched” is the highest and the number of “Not Matched” is higher than normal LDA, but lesser than the other two methods, SignatureAnalyzer and SigProfiler. In particular, SBS6 and SBS7-a can be extracted only with PLDA, which is expected to predict signatures specific to the tumor types for which only a small number of samples are available (here, it corresponds to 6th and 7th artificial tumor types) with real mutation catalogs.

Results of the simulation experiments with artificial mutation catalogs following PLDA generation process indicated that PLDA and SignatureAnalyzer showed the best performance in model selection, and predicted the correct signature sets without any errors in general cases. In addition, for special cases where signature estimation is difficult, PLDA outperformed the existing methods, including SigProfiler and SignatureAnalyzer.

### 3.2. Simulation with PCAWG Synthetic Data

As described in Section 3.1, PLDA performs comparably to existing methods when using artificial data that follows the generation process of PLDA. In this section, we examined whether PLDA performs equally well on synthetic mutation catalogs generated from signatures predicted by SigProfiler. We applied PLDA to the synthetic mutation catalogs containing 1350 samples published by PCAWG project (https://www.synapse.org/#!Synapse:syn18930922) to predict signatures. This mutation catalog consists of nine tumor types (each tumor type has 150 samples), and ground-truth signature compositions and the decomposition results can be utilized by applying SignatureAnalyzer or SigProfiler. Further, the signature activities of these synthetic data are determined by a process that mimics a realistic cancer evolution, rather than being sampled from a pure probabilistic distribution such as Dirichlet distribution. To verify the effectiveness of the proposed method, we compared the result of PLDA against ground-truth and other methods.

First, this mutation catalog is composed of 21 signatures determined by SigProfiler, and PLDA predicted K=19 signatures. Table 7 shows the matching results based on the cosine similarity when predicted signatures were matched to the correct signatures and vice versa. From this table, it can observed that some of the signatures predicted by PLDA match the same true signature (e.g., both of predicted 1 and 17 match SBS2), and PLDA could not extract five true signatures (SBS21, SBS28, SBS29, SBS30, and SBS9). This trend is consistent with the results of signature prediction using SigProfiler. SigProfiler predicted 16 signatures, and all of them identically matched to the correct signatures. Moreover, the correct signature sets missed by SigProfiler were exactly the same as PLDA. On the contrary, SignatureAnalyzer could extract 19 unique signatures but missed SBS29 and SBS9.

Using synthetic mutation catalogs, we observed that the signature sets predicted by PLDA missed some of the ground-truth signatures as well as the other methods; however, this is an inevitable problem with decomposition-like approaches (including NMF). To evaluate the result of decomposition, we define Reconstruction Rate, RR, as follows:

(2) $$\begin{array}{c} RR=\frac{1}{L}\sum_{l=1}^{L}\frac{1}{S_{l}}\sum_{s=1}^{S_{l}}\frac{1}{n_{ls}}\sum_{v=1}^{V}\min\left(M_{lsv},n_{ls}\times {\left[\theta_{ls}\phi \right]}_{v}\right) \end{array}$$

where $M_{lsv}$ and $n_{ls}\times {\left[\theta_{ls}\phi \right]}_{v}$, respectively, show the element of input matrix of mutation catalog and its approximated value from the predicted parameter of the *v*th mutation type in *s*th sample with *l*th tumor type. RR has a value ranging from 0.0 to 1.0, and RR=1.0 indicates that the original mutation catalog could be completely reconstructed. The calculated RR value in PLDA was RR=0.9895, which was considerably high, and was comparable to other methods (SigProfiler:0.9949, SignatureAnalyzer: 0.9018). We have summarized these results in Table 8. It is noteworthy that RR value is not an objective function maximized directly by PLDA, but is a criterion by which all methods can be evaluated equally. Therefore, a solution with a sufficiently high RR value that is different from ground-truth is one of the local-minimums, and the experiments should be repeated multiple times with different initial values of parameters to avoid such solutions. In this simulation, PLDA, SigProfiler and SignatureAnalyzer were run 50, 20 and 20 times, respectively; however, due to high computational costs involved, it is impossible to repeat enough to avoid the local-minimum. In addition, the signature compositions with high RR value indicate that the contribution of missed signatures with several approaches (PLDA and SigProfiler) could be sufficiently explained by other signatures.

In addition to the data sets listed here, we applied PLDA to two PCAWG datasets (one is the large whole-genome sequenced mutation catalog and the other is the whole-exome sequenced mutation catalog across multiple tumor types) and have presented the results and the comparisons against the existing methods in Supplementary Section S4. Taken together, we have extracted similar signatures as predicted by other methods, for the dataset described here. Particularly in whole-exome sequenced data, PLDA extracted more number of correct signatures than SigProfiler and showed a higher reconstruction rate than SigProfiler and SignatureAnalyzer.

### 3.3. Real Data Analysis

**Figure 3:**
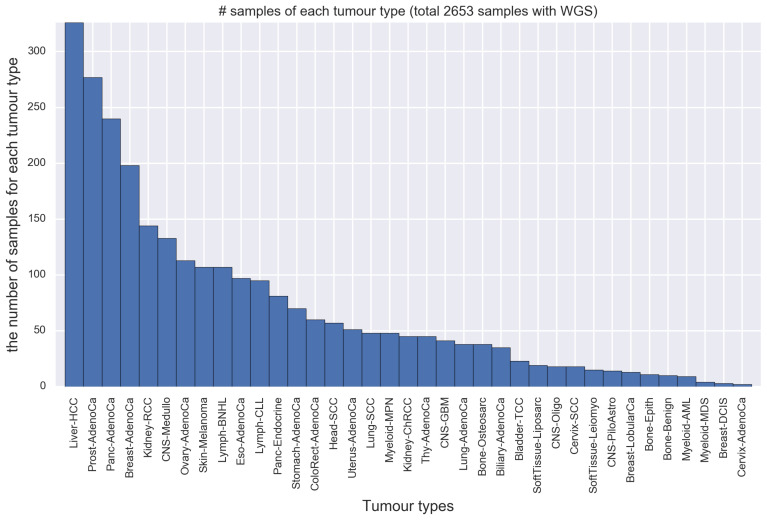
The number of samples contained in each tumor type. The horizontal axis represents each tumor type, and the vertical axis represents the number of samples corresponding to the tumor type in the mutation catalog of whole-genome sequenced (WGS). The tumor type with the most samples is Liver-HCC (326 samples), while that with the least samples is Cervix-AdenoCa (2 samples).

As an actual mutation catalog, we used whole-genome variants calls produced by ICGC/TCGA Pan-Cancer Analysis of Whole Genomes (PCAWG). Their original resources are available at online (https://www.synapse.org/#!Synapse:syn11726616). The original data contains 2780 samples for single nucleotide substitution (SBS) counts. We removed samples with few mutations (less than 400 mutations) as a preprocessing because the presence of samples with low-variant interferes with learning in LDA-based approaches [16], and we obtained 2653 samples. Figure 3 shows the number of samples contained in each tumor type. This mutation catalog consists of 37 tumor types.

Then, we applied PLDA to actual mutation catalogs. With the model selection, the number of signatures included in the mutation catalog was predicted to be K=41. Detailed results of model selection are shown in Supplementary Section S5 and Supplementary Figure S2. Table 9 shows the results of matching 41 predicted signatures with COSMIC v3.1 signatures based on cosine distance of mutational distribution (concrete cosine distances between all predicted signatures and COSMIC v3.1 signatures are detailed in Supplementary Table S5). Out of 49 known signatures considered to exist, predicted signatures were mapped to 30 types of signatures. In addition, for some known signatures, multiple predicted signatures were mapped (e.g., for SBS7a, SBS10a, SBS11, SBS17b, SBS21 and SBS28), and these predicted signatures will be discussed later in Section 4.1. Compared with the artificially created mutation catalogs according to the ideal generation process, the actual mutation catalog is biased by various factors. Therefore, we think some possible local minimum solutions exist, and that it is difficult to reconstruct exact identical signatures predicted by other methods. Further, all of these known signatures were predicted by SigProfiler (NMF-based approach), and it is natural that the signatures predicted by LDA-based approach (i.e., PLDA) are different. Another reason may be that samples with extremely low mutation numbers were removed by the preprocessing in this experiment. Based on these results, the important known signatures (such as SBS3) that could not be extracted by PLDA are further discussed in Section 4.2.

**Figure 4:**
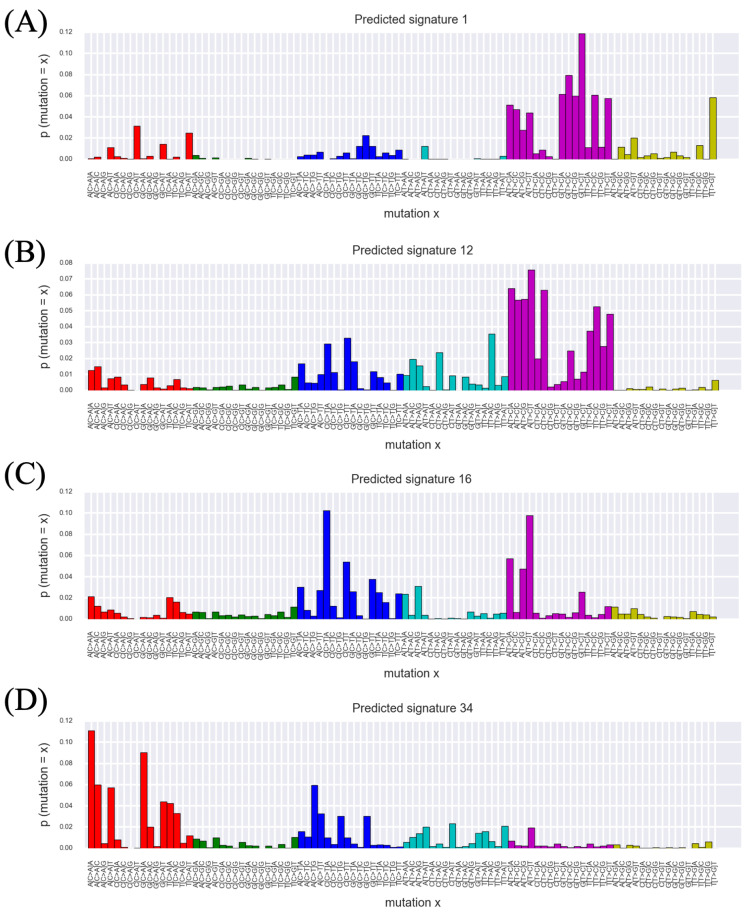
A part of the signatures predicted by PLDA. Each panel of (**A**–**D**) shows the mutational distribution of a signature predicted by PLDA. The horizontal axis shows the types of mutations, which include the type of substitution and the base before and after the substituted base, and the vertical axis shows the mutational probability. (**A**–**D**) shows Predicted Signatures 1, 12, 16 and 34, and these were mapped to SBS21, SBS12, SBS5 and SBS29 with cosine distances = {0.261,0.204,0.233,0.255}, respectively. These signatures are commonly active at **CNS-Oligo** ([Central Nervous System]-[Oligodendroglioma]) samples. (**A**) This signature has peaks mainly at A[T>C]X and G[T>C]X, and also at X[C>A]T and T[T>G]T. (**B**) This signature has peaks mainly at A[T>C]X and T[T>C]X, and also at some of [C>T] and [T>A] substitutions. (**C**) This signature has peaks at X[C>T]A and A[T>C]X. (**D**) This signature has peaks at some of [C>A] substitution.

**Figure 5:**
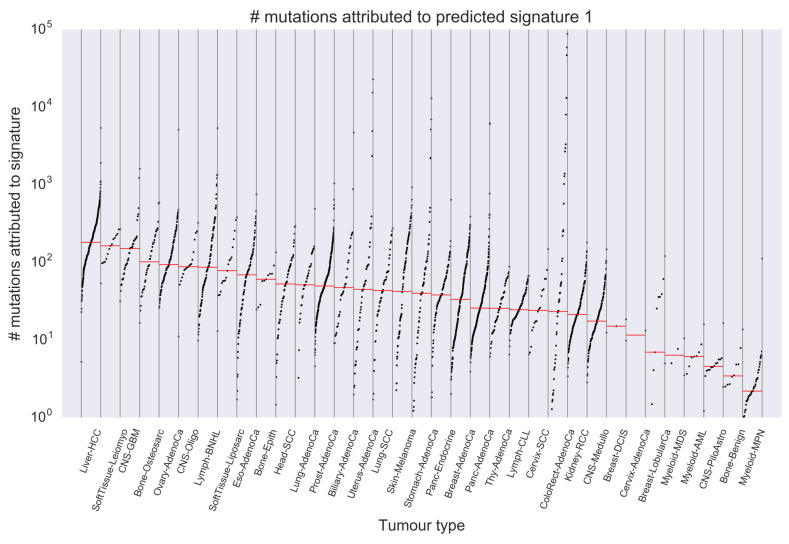
The number of mutations attributed to Predicted Signature 1. This figure shows the number of mutations attributed to Predicted Signature 1 for each sample by tumor types. The horizontal axis shows the tumor types, and the vertical axis shows the number of mutations derived from Predicted Signature 1. Each plot shows that of each sample, and red bar shows median value among them. From this figure, we can see that Predicted Signature 1 is active across many tumor types, and consider that it is unlikely to be an artifact active in only a few samples. We have performed the same visualization for Predicted Signatures 12, 16 and 34 (listed in Figure 4), and found that the number of mutations attributed to them are commonly high in samples from Liver-HCC, CNS-Oligo and so on.

In the latter part of this section, we show that the interpretation of the mutation catalog reconstructed by the signatures predicted by our method provides different landscapes compared to using the known signatures. Figure 4 shows mutational distributions of Predicted Signatures 1, 12, 16 and 34. In the mapping to known signatures described in the previous paragraph, these signatures were mapped to SBS21, SBS12, SBS5 and SBS29 with cosine distances = {0.261,0.204,0.233,0.255}, respectively, but these values are too large to consider that the matched known signatures and the predicted signatures are the same. From Figure 4, we can see that each of these signatures has characteristic peaks depending on adjacent bases of mutated base. However, it is difficult to judge whether or not these signatures are undiscovered existing signatures only by in silico experiments. Next, we investigated in which tumor type these signatures are active. Figure 5 shows the number of mutations attributed to Predicted Signature 1 for each tumor type. The expected value of the number of mutations $\xi_{lsk}$ attributed to the *k*th signature in the *s*th sample from the *l*th tumor type with PLDA can be calculated as follows:

(3) $$\begin{array}{c} \xi_{lsk}=n_{ls}\times \theta_{lsk}. \end{array}$$

**Figure 6:**
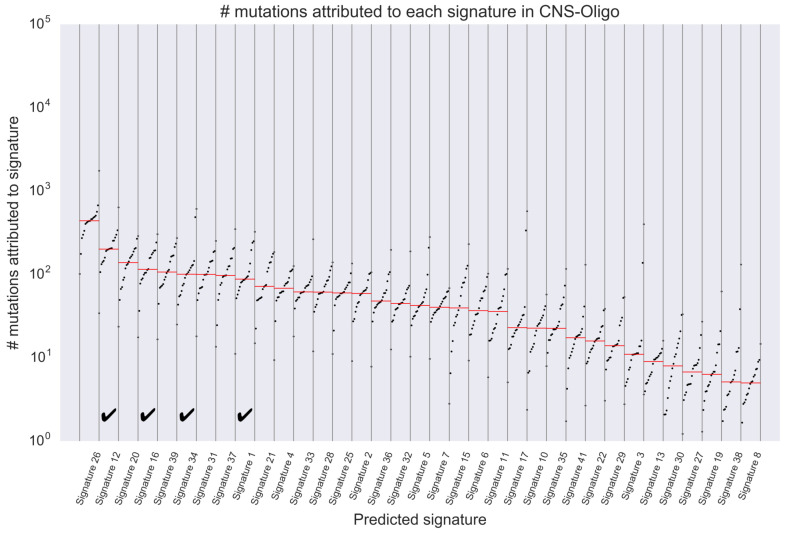
The number of mutations attributed to each signature with samples from CNS-Oligo. This figure shows the number of mutations attributed to each predicted signature in samples from CNS-Oligo, and can be interpreted in a similar manner to Figure 5. The checked columns show the signatures of interest, which are listed in Figure 4 and they have large cosine distances to the most closely known signatures. This figure indicates that the signature of interest has large contributions to the mutations of almost samples from CNS-Oligo.

From this figure, we can see that Predicted Signature 1 is active across many tumor types, and consider that it is unlikely to be an artifact active in only a few samples. We have performed the same visualization for Predicted Signatures 12, 16 and 34 (listed in Figure 4, and these visualization results are seen in Supplementary Figures S3–S5), and found that the number of mutations attributed to them are commonly high in samples from Liver-HCC (Hepatocellular carcinoma), CNS-Oligo (Central Nervous System-Oligodendroglioma) and so on. Since the number of samples from CNS-Oligo is $S_{l}=18$, which is small sample size and makes analysis difficult by the previous method, it is noteworthy that our method could predict the signature distribution in a similar manner to that from liver. In addition, Figure 6 shows the number of mutations attributed to each predicted signature in samples from CNS-Oligo, and this figure indicates that the signature of interest has large contributions to the mutations of most samples from CNS-Oligo. Compared with the same visualization of Liver-HCC (see Supplementary Figure S6), it was again found that the signatures that highly contributed to mutations in CNS-Oligo and Liver-HCC were common. Furthermore, we can see that the signature that contributed most to mutations in CNS-Oligo was the Predicted Signature 26 from Figure 6, and that this signature has a similar mutational distribution to SBS1 (related with 5-methylcytosine deamination) with cosine distance = 0.008. Previous analyses of activity found that SBS1 was active in the CNS-Oligo sample (https://cancer.sanger.ac.uk/cosmic/signatures/SBS/SBS1.tt), leading to consistent results with this experiment. In the previous studies, the active signatures in the sample obtained from CNS-Oligo were considered to be SBS 1, 5, 8 and 40 [11]. Among these known signatures, SBS5 and SBS40 have slightly similar distributions to Predicted Signatures 16 and 2 (both of them have much contributions to mutation in CNS-Oligo) with cosine distance = {0.233,0.166}, respectively, which reinforces the reliability of this result. However, PLDA did not yield a signature that matched to SBS8. This result may suggest that the mutations that were supposed to attribute to SBS8 are deconvoluted by signatures predicted by our method (e.g., Predicted Signature 1, 12, and 34). It is difficult to provide evidence that positively supports such hypotheses in practice; however, it is worth noting that our approach provides different view of interpretation of observed mutation catalogs other than that shown in previous studies.

## 4. Discussion

### 4.1. Difficulty to Predict Signatures with Some Tumor Types

**Figure 7:**
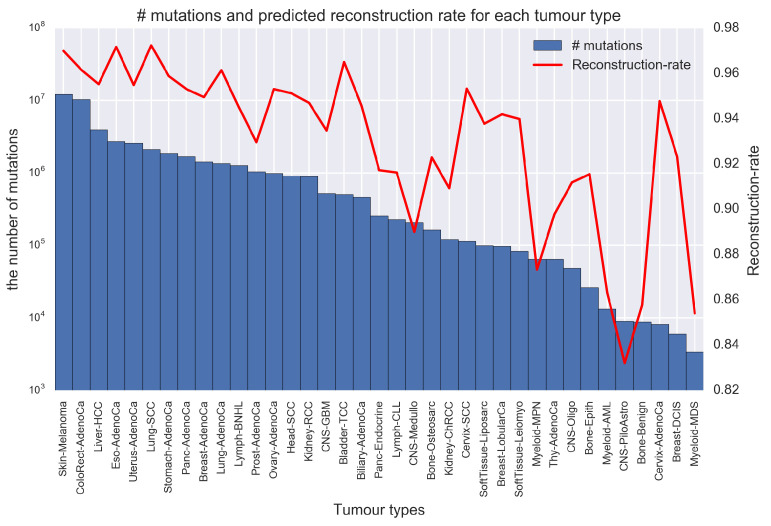
The number of all mutations and reconstruction rate for each tumor type. This figure shows the number of all mutations (blue bar) and reconstruction rate (red-line) for each tumour type. The horizontal axis shows each tumor type, and the two vertical axes show the number of mutations (note that this axis is log-scaled) and reconstruction rate, respectively. The reconstruction rate indicates the average value of how much the observed mutation is explained by the predicted model, and higher reconstruction rate indicates better reconstruction of the original mutation catalog.

The proposed method has enabled to provide new landscapes of signatures introduced in the previous section; however, certain limitations remain. From Table 9, we can see that multiple signatures matched to the same known signature (e.g., SBS7a). Supplementary Figure S7 shows the Predicted Signatures 19, 23, 27 and 29, which are matched to SBS7a. All have peaks at T[C>T]X and they differ in some other mutations. In addition, all of these signatures were extremely active in samples from Skin-Melanoma. These signatures were predicted through appropriate model selection and are not considered to be unambiguous duplicate errors (again see Supplementary Section S5 for the model selection of PLDA). However, obtaining multiple similar signatures like these results may reduce interpretability, and we want to avoid such phenomenon as possible as we can. Then, we conducted further analysis and found that the cause of this problem was related to the number of all mutations per tumor type. Figure 7 shows the number of mutations and reconstruction rate for each tumor type. The reconstruction rate for *l*th tumor type, $RR_{l}$, is the average value of how much the observed mutation is explained by the predicted model among samples belonging to *l*th tumor type, and it is calculated for each tumor type for the RR defined in Equation (2). $RR_{l}$ takes from 0.0 to 1.0, and $RR_{l}=1.0$ indicates that the original mutation catalog could be completely reconstructed for *l*th tumor type. Figure 7 indicates that tumor type with a large number of mutations has consistently high reconstruction-rates, and some tumor types with a small number of mutations have low reconstruction-rates compared with higher ones. This result suggests that, in the process of learning parameters, PLDA prioritize to deconvolute samples from a tumor type with a large number of mutations (even if the error at other tumor types becomes large) because the error of samples from such a tumor type greatly contributes to the objective function (variational lower bound). Therefore, in tumor type with many mutations, such as Skin-Melanoma, PLDA predicts multiple "high-resolution" signatures with slightly different mutational distributions (e.g., Predicted Signatures 19, 23, 27 and 29) in order to reduce the total errors, and these extractions lead to decrease in the reconstruction rate of samples from some tumor type with a small number of mutations.

However, this property of PLDA is not the only drawback. As introduced in Section 3.3, we found that some predicted signatures in CNS-Oligo are difficult to associate with known signatures, and they are also active in Liver-HCC. The number of all mutations and reconstruction-rate in Liver-HCC are high, and it is considered that PLDA predicts multiple high-resolution signatures for samples from Liver-HCC. Despite the small number of mutations in CNS-Oligo, it is worth noting that the mutation catalogs of CNS-Oligo are well-reconstructed ($RR_{l}=0.912$) from a subset of signatures that are predicted in concert with the Liver-HCC catalogs. There are other relationships between tumor types like Liver-HCC and CNS-Oligo. Although the number of mutations of Cervix-AdenoCa (Adenocarcinoma) is very small, that reconstruction is good ($RR_{l}=0.948$) using predicted signatures active in ColoRect-AdenoCa (Colorectal-Adenocarcinoma), Bladder-TCC (Transitional Cell Carcinoma), and so on.

### 4.2. Some Important Signatures Could Not Be Extracted by PLDA

As described in Section 3.3 and Section 4.1, some signatures reported in COSMIC v3.1 could not be extracted by using PLDA because our method differs from the conventional NMF-based approaches in the generation process. Particularly, some of the known signatures have not been predicted by our method, although their existence has been experimentally confirmed. Such results may impair the reliability of the proposed method, therefore, such signatures are discussed here individually.

First, SBS3 is believed to be associated with defective homologous recombination-based DNA damage repair, but PLDA was unable to extract it. We presume that Predicted Signature 2 that matches SBS40, which has a similar distribution to SBS3 (cosine distance between SBS3 and SBS40 is 0.118) is related to it. Supplementary Figure S8 shows the mutational distributions of Predicted Signatures 2, 17 (which matches SBS3 with cosine distance = 0.298), SBS3 and SBS40. It can be observed that the Predicted Signature 2 has a similar mutational distribution to SBS3, and its cosine distance (0.195) is small. In addition, Supplementary Figure S9 shows the number of mutations attributed to Predicted Signature 2 for each tumor type. SBS40 is unlikely to be active in breast cancer samples, but our results showed that Predicted Signature 2 is active in breast samples. SBS3 is likely to be activated in the BRCA-deficient breast cancer samples, and since Predicted Signature 2 too is active in breast cancer samples, we speculate that both were extracted as one signature mixture composed of SBS3 and SBS40. The etiology of SBS40 is unclear and we do not know whether this signature really exists, but it is noteworthy that it is difficult to classify signatures like SBS3 and SBS40, which have very similar mutational distributions. In this experiment, several such results were observed in addition to SBS3. For example, Predicted Signature 15 matches SBS19 with cosine distance = 0.105, but it also shows a spectrum similar to SBS31, which could not be extracted (cosine distance is 0.128, see Supplementary Table S5).

Next, SBS35 is associated with prior chemotherapy treatment with platinum drugs, and PLDA could not extract this signature, although this has been experimentally validated in multiple systems [26]. In signature extraction using PLDA, Predicted Signature 3 has the closest distribution to SBS35, but their cosine distance is 0.357, which is rather large (see Supplementary Table S5). SBS35 is mainly active in a few tumor samples including ten Liver-HCC samples and one Ovary-AdenoCa (Adeno Carcinoma) sample of PCAWG-WGS data (https://cancer.sanger.ac.uk/cosmic/signatures/SBS/SBS35.tt). As mentioned in Section 4.1, PLDA can miss signatures that are active only in a few samples, and SBS35 is a typical example of such a signature. The same is true for SBS24 which is associated with exposures to aflatoxin [27]. In a previous study [11], the researchers used the whole-genome sequenced catalogs of PCAWG project to determine the mutational distributions of the signatures, and then re-estimated the activity including data obtained from the other projects and whole-exome sequenced data. As a result, SBS24 was found to be active in only two samples (one Biliary-AdenoCa and one Liver-HCC samples) of the PCAWG-WGS data used to determine the mutational distribution (https://cancer.sanger.ac.uk/cosmic/signatures/SBS/SBS24.tt). As shown in Figure 7, Liver-HCC, which is prone to activated SBS24 and SBS35, has a high reconstruction rate with PLDA, and therefore, its mutation catalog is well explained by our signature sets. However, when using PLDA, we need to be aware that some signatures such as those discussed here may be missed.

Finally, we provide a plausible explanation for the signatures that have similar mutational distributions as ultraviolet light-related signatures, SBS7-c and SBS7-d, and were not extracted by PLDA. These have been reported in recent studies, and by COSMIC version 2 only Signature 7 was considered to be associated with ultraviolet light (https://cancer.sanger.ac.uk/cosmic/signatures_v2.tt). The availability of more datasets may explain the discovery of these new signatures. In general, Skin-Melanoma samples where UV-light related signatures are more likely to be active have a much higher number of mutations than the other tumour types. As shown in Section 4.1, such an imbalance in the number of mutations is likely to hinder the analysis, and previous methods including SignatureAnalyzer were unable to overcome this problem. For example, de novo signature extraction using SignatureAnalyzer differentially predicted the signatures for Skin-Melanoma samples with more mutations, and the other tumors [11]. In the same study, signature extraction from hypermutated samples presented different signature profiles between methods (SigProfiler and SignatureAnalyzer) even when using synthetic data. Based on these observations, we hypothesize that it is difficult to predict signatures such as SBS7-c and SBS7-d, even if there are many mutations or samples that contribute.

So far, we have provided a plausible explanation for why PLDA missed some important signatures. The available scientific information as well as results of our study present two possible directions for signature prediction in future. First, the need for a complete de novo prediction of mutational distributions may disappear gradually. This is because the concept of mutation signatures and the certainty of the mutational distributions of some signatures are being verified by various experiments in vivo/vitro as described in the Introduction section, and it will become unnecessary to re-estimate the mutational distributions of these signatures only from the mutation catalog. In such a scenario, there is a need for a technology that can accurately predict signature activities from subset of mutation catalogs, and we believe that the idea of PLDA will be a key player in this field. In fact, even after the current signature sets were determined, new signatures have been reported using a subset of the mutation catalog by the same method (SigProfiler, for example) [28,29], highlighting the necessity to discover new signatures while considering the balance with the validated signatures. In this study, it was not possible to separate signatures with similar mutational distributions such as SBS3 and SBS40. However, if the presence of SBS40 is experimentally validated, PLDA can easily address such problems by fixing the mutational distributions of these signatures and re-estimating their activities. Furthermore, PLDA controls the activity of each tumor type using different hyperparameters, and therefore, we believe that PLDA can be applied to a subset across tumor types, which may lead to the discovery of some subset-specific signature. Another direction, though a very difficult task, is to construct a pipeline for fully de novo signature extraction, including hypermutated samples without any ad hoc method. As the signatures corresponding to SBS7-c and SBS7-d were not obtained in this study, we speculate that signature prediction in hypermutated samples is a difficult task. To the best of our knowledge, all of the previous methods require manual determination of hyperparameters or partitioning of data sets, and we think that a pipeline that can extract signatures without the need for an ad hoc criterion would be a reliable method from the viewpoint of mathematical validity.

### 4.3. Perspective

In Section 3.3, we showed that the CNS-Oligo mutation catalogs, which has only a small number of samples ($S_{l}=18$), can be explained differently than the previous method by using a set of predicted signatures with our method. Here, we further discuss its justification, and show significance of the existence of our method. In the first place, attempts to explain mutation catalogs from the tumor types with a small number of samples or mutations by a large number of signatures can always lead to an ill-posed problem. In a simple example, when the number of signatures *K* is larger than the number of samples $n_{ls}$, by constructing signature ϕ such that each signature $\phi_{k}$ represents the ratio of mutational burden in each sample, the original mutation catalog can be completely expressed without any deconvolution. These facts indicate that there are multiple ways (in other words, singular solutions) of expressing a mutation catalog by signatures, and it is difficult to compare them; that is, it is difficult to determine the most effective method for any mutation catalog, in principle. In this study, though PLDA and SigProfiler yield the similar results when using artificial mutations in simulation experiment, these methods predict different signature sets with the actual mutation catalogs, which also shows the difficulty in determining the method. The most important thing for us to understand the carcinogenic mechanism through the mutation signature analyses is to clarify its etiology. Unfortunately, to our knowledge, no etiology was considered to be related with our predicted signatures which have different mutational distributions from known signatures. In addition, previous studies have revealed many of the signatures, but there are many ones that have not been revealed corresponding etiology. In this situation, it is important for connecting with aetiology that there are multiple ways of interpreting mutations by signatures, we believe. This is because, for example, when a new etiology contributing to carcinogenesis is found, using a method that can obtain the signature corresponding to it may lead the elucidation of new factors as other signatures.

As another important study understanding the relationship between the predicted mutation signature and carcinogenic mechanism, it is possible to investigate the relationship between mutation signatures and survival time. For example, if there is a survival time to be analyzed in the metadata of the sample (individual) and it can be estimated from the predicted signature activity by a method such as regression, we can find the signature related to the mortality rate (i.e., mutational processes involved in disease progression) by referring to the regression coefficient. In addition, from a therapeutic perspective, it may be useful to research the relationship between tumor mutational burden (TMB) and mutation signatures. It is widely known that immune checkpoint inhibitors are often effective in patients with high TMB levels [30,31]. In the future, investigation of a correlation between a particular signature activity and TMB will enable us to use the signature as a guideline to precision medicine. Thus, by combining the predicted signature activity with other metadata of samples, new insights may be discovered.

## 5. Conclusions

This study proposed PLDA, an extended model of LDA, and developed a method for predicting mutation signatures without dividing the mutation catalog by tumor types. In our method, merging signatures via post-analysis such as clustering is no longer necessary. We have confirmed that proposed model can predict signatures with the almost same accuracy as previous methods (including SigProfiler and SignatureAnalyzer) through simulation experiments using multiple synthetic mutation catalogs including the catalog that mimic synthetic scenario emulating the PCAWG pan-tissue data set. Furthermore, we applied PLDA to actual mutation catalogs of PCAWG, and obtained a different signature set from the previous method (SigProfiler). Although some important signatures were missed by PLDA, we have provided plausible explanations for them, as well as highlighted the future directions for mutation signature extraction. From this experiment, PLDA shows that the reconstruction of the mutation catalog using our predicted signatures gives us a novel interpretability when looking at mutation spectrum.

## Figures and Tables

**Table 1:** Simulation setting for generating artificial data.

Tumor Type	SBS1	SBS2	SBS3	SBS4	SBS5
1st	∘	∘	×	×	×
2nd	×	∘	∘	×	×
3rd	×	×	∘	∘	×
4th	×	×	×	∘	∘
5th	∘	×	×	×	∘

This table shows which signature appears at which tumor type when generating artificial data for simulation. In each cell, circles indicate that the signature is likely to appear ($\alpha_{lk}=0.5$); conversely, crosses indicate that it is difficult to appear ($\alpha_{lk}=0.05$). From the property of Dirichlet distribution, the signatures with circles are 10 times more active than those with crosses.

**Table 2:** Comparison of model selection performance between PLDA and the previous methods with a simulation for artificial data.

Methods\$K_{\mathrm{predicted}}$	2	3	4	5 (True)	6	7	8	Total
PLDA (Proposed)	0	0	0	**100**	0	0	0	100
SignatureAnalyzer	0	0	0	**100**	0	0	0	100
SigProfiler	0	0	91	**8**	1	0	0	100
Normal LDA	0	100	0	**0**	0	0	0	100

As shown in Table 1 and Section 3.1, we generated artificial mutation catalogs 100 times, and compared whether each method could accurately predict the number of signatures. This table shows how many times the number of signatures was selected ($K_{\mathrm{predicted}}$) out of 100 times by each method. As the correct number of signatures is five (bold text in the table), the prediction was performed accurately in the order of PLDA (proposed) = SignatureAnalyzer, SigProfiler, and normal LDA.

**Table 3:** Matching results of predicted signatures with ground-truth.

Method	SBS1	SBS2	SBS3	SBS4	SBS5
PLDA (Proposed)	100	100	100	100	100
SignatureAnalyzer	100	100	100	100	100
SigProfiler	100	100	9	100	100
Normal LDA	100	100	98	0	0

Method	Correct Matched	Duplicated	Not Matched	Total
PLDA (Proposed)	500	0	0	500
SignatureAnalyzer	500	0	0	500
SigProfiler	409	0	1	410
Normal LDA	298	0	2	300

This table shows the matching results based on the cosine distance when the signatures predicted by each method are matched to the correct signatures. The upper table shows the number of times each correct signature was predicted (threshold of cosine distance is 0.1), and the lower table shows the summary. In the lower table, “Duplicated” column shows the number of predicted signatures which match the same correct signatures. “Not Matched” column also shows the number of predicted signatures which did not match any of the correct signatures, and these columns indicate the number of false positives.

**Table 4:** The simulation scenario for generating artificial data with difficult settings to predict signatures.

Tumor Type	SBS1	SBS2	SBS3	SBS4	SBS5	SBS6	SBS7-a	SBS7-b	SBS7-c	SBS7-d
1st	∘	∘	×	×	×	×	×	×	×	×
2nd	×	∘	∘	×	×	×	×	×	×	×
3rd	×	×	∘	∘	×	×	×	×	×	×
4th	×	×	×	∘	∘	×	×	×	×	×
5th	∘	×	×	×	∘	×	×	×	×	×
6th	×	×	×	×	×	∘	×	×	×	×
7th	×	×	×	×	×	×	∘	×	×	×
8th	×	×	×	×	×	×	×	∘	×	×
9th	×	×	×	×	×	×	×	×	∘	×
10th	×	×	×	×	×	×	×	×	×	∘

This table shows which signature appears for which tumor type when generating artificial data for simulation using difficult setting to predict signatures. This table can be interpreted in manner similar for Table 1. Compared with the case of Table 1, five signatures (SBS6, SBS7-a, SBS7-b, SBS7-c, SBS7-d) are added, and each signature is set to be more active in the five newly added virtual tumor types (6th∼10th). In addition, all the added tumor types mimic a situation where the sample size is small ($S_{l}=2$).

**Table 5:** Comparison of model selection for artificial data with difficult settings to predict signatures.

Methods \ $K_{\mathrm{predicted}}$	2	3	4	5	6	7	8	9	10 (true)	11	12	Total
PLDA (Proposed)	0	0	0	0	0	0	1	72	**26**	1	0	100
SignatureAnalyzer	0	0	0	0	0	0	0	93	**7**	0	0	100
SigProfiler	0	0	0	0	0	7	26	29	**38**	0	0	100
Normal LDA	0	100	0	0	0	0	0	0	**0**	0	0	100

According to the settings of Table 4, artificial mutation catalogs were generated 100 times, and compared to elucidate whether each method could accurately predict the number of signatures. This table can be interpreted in a similar manner to Table 2.

**Table 6:** Matching results of predicted signatures with ground-truth in the difficult cases.

Method	SBS1	SBS2	SBS3	SBS4	SBS5	SBS6	SBS7-a	SBS7-b	SBS7-c	SBS7-d
PLDA (Proposed)	100	100	95	100	96	79	22	98	100	100
SignatureAnalyzer	100	100	99	100	64	0	0	97	100	100
SigProfiler	100	100	46	100	0	0	0	35	100	100
Normal LDA	100	100	89	0	0	0	0	0	0	0

Method	Correct Matched	Duplicated	Not Matched	Total
PLDA (Proposed)	890	0	37	927
SignatureAnalyzer	760	0	147	907
SigProfiler	581	0	317	898
Normal LDA	289	0	11	300

This table shows the matching results based on the cosine distance when the signatures predicted by each method are matched to the correct signatures in the difficult cases (threshold of the cosine distance is 0.1). This table can be interpreted in a similar manner to Table 3.

**Table 7:** Simulation result of PLDA applying to PCAWG synthetic mutation catalogs.

Predicted	Matched	Cosine Similarity	True	Matched	Cosine Similarity
1	**SBS2**	0.7780	SBS1	15	1.0000
2	SBS13	0.9987	SBS13	2	0.9987
3	**SBS5**	0.8729	SBS15	13	0.9946
4	SBS3	0.9155	SBS17a	14	0.9999
5	SBS26	0.9691	SBS17b	7	1.0000
6	**SBS5**	0.8792	SBS18	**9**	0.9933
7	SBS17b	1.0000	SBS2	17	0.9999
8	SBS22	0.9858	SBS21	**5**	0.7372
9	SBS18	0.9933	SBS22	8	0.9858
10	SBS41	0.9856	SBS26	**5**	0.9691
11	SBS40	0.7277	SBS28	**11**	0.7042
12	SBS4	0.9308	SBS29	**9**	0.8110
13	SBS15	0.9946	SBS3	4	0.9155
14	SBS17a	0.9999	SBS30	**6**	0.7484
15	SBS1	1.0000	SBS4	12	0.9308
16	SBS8	0.9320	SBS40	16	0.7617
17	**SBS2**	0.9999	SBS41	10	0.9856
18	SBS44	0.9446	SBS44	18	0.9446
19	**SBS5**	0.8762	SBS5	**6**	0.8792
-	-	-	SBS8	16	0.9320
-	-	-	SBS9	**11**	0.7027

This table shows the matching results based on the cosine similarity when predicted signatures were matched to the correct signatures (left three columns) and vice versa (right three columns). Signatures with bold letters are the ones that could not be matched one-to-one, and some correct signatures with underlines indicate the ones that were not extracted by PLDA. Therefore, PLDA could extract 16 identical signatures.

**Table 8:** Comparison of the methods with synthetic WGS mutation catalogs from PCAWG project.

Method	# Extracted (True:21)	Avg. Cosine Similarity	Reconstruction Rate
PLDA (proposed)	16	0.9194	0.9895
SigProfiler	16	0.9387	0.9949
SignatureAnalyzer	19	0.9642	0.9018

This table shows the comparison of the methods with synthetic WGS mutation catalogs published from PCAWG project. “# Extracted”, “Avg. Cosine Similarity” and “Reconstruction Rate” columns show the number of extracted correct signatures (removing duplicated matched signatures), average cosine similarity of all matches calculated from Table 7 and reconstruction rate calculated from Equation (2), respectively.

**Table 9:** Mapping the signatures obtained with PLDA to known signatures from COSMIC v3.1.

SBS1	SBS2	SBS3	SBS4	SBS5	SBS6	SBS7a	SBS7b	SBS7c	SBS7d
∘	∘	×	∘	×		∘∘∘×	∘		
SBS8	SBS9	SBS10a	SBS10b	SBS11	SBS12	SBS13	SBS14	SBS15	SBS16
		∘∘∘▵	∘	∘×	×	∘	∘	∘	∘
SBS17a	SBS17b	SBS18	SBS19	SBS20	SBS21	SBS22	SBS23	SBS24	SBS25
∘	∘×	∘	▵		∘×	∘			
SBS26	SBS27	SBS28	SBS29	SBS30	SBS31	SBS32	SBS33	SBS34	SBS35
▵		∘∘	×					▵	
SBS36	SBS37	SBS38	SBS39	SBS40	SBS41	SBS42	SBS43	SBS44	SBS45
	×		▵	▵		×		×	
SBS46	SBS47	SBS48	SBS49	SBS50	SBS51	SBS52	SBS53	SBS54	SBS55
							×		
SBS56	SBS57	SBS58	SBS59	SBS60	SBS84	SBS85			
									

This table shows the results of mapping 41 signatures predicted via PLDA to the known signatures from COSMIC v3.1 with the closest cosine distance. Circles, triangles, crosses indicate that cosine distance between a predicted signature and matched known signature is smaller than 0.1, 0.2 and larger than 0.2, respectively. For some known signatures, multiple predicted signatures were mapped (e.g., for SBS7a). Concrete cosine distances between all predicted signatures and COSMIC v3.1 signatures are detailed in Supplementary Table S5.

## Supplementary Materials

The following are available online at https://www.mdpi.com/2073-4425/11/10/1127/s1, Figure S1: The graphical model of Supervised LDA, Figure S2: Model selection of PLDA with real mutation catalogs, Figure S3: The number of mutations attributed to Predicted signature 12, Figure S4: The number of mutations attributed to Predicted signature 16, Figure S5: The number of mutations attributed to Predicted signature 34, Figure S6: The number of mutations attributed to each signature with Liver-HCC, Figure S7: Predicted signature 19, 23, 27, 29, and SBS7a, Figure S8: Predicted signature 2, 17, SBS3 and SBS40, Figure S9: The number of mutations attributed to Predicted signature 2, Table S1: Matching result of synthetic large WGS data published from PCAWG project, Table S2: Comparison of the methods with synthetic large WGS data Method, Table S3: Matching result of synthetic WES data published from PCAWG project, Table S4: Comparison of the methods with synthetic WES data, Table S5: Matching result with real data published from PCAWG project.

## Abbreviations

The following abbreviations are used in this manuscript:

NMF	Non-negative Matrix Factorization
LDA	latent Dirichlet allocation
PLDA	Parallelized latent Dirichlet allocation
PCA	principal component analysis
*t*-SNE	*t*-distributed Stochastic Neighbor Embedding
SBS	Single Base Substitution
PCAWG	Pan Cancer Analysis of Whole Genomes
RR	Reconstruction Rate
CNS	Central Nervous System
HCC	Hepatocellular Carcinoma
Oligo	Oligodendroglioma
AdenoCa	Adeno Carcinoma
