# Supplementary material for: Parallelized Latent Dirichlet Allocation Provides a Novel Interpretability of Mutation Signatures in Cancer Genomes
Source: Genes (Basel). 2020 Sep 25;11(10):1127. doi: 10.3390/genes11101127 (PMC7600398; doi:10.3390/genes11101127)
Supplement: Supplementary file 1 [file genes-11-01127-s001.zip › submission_Genes_suppl_final/suppl_final.pdf]

Supplementary Information for "Parallelized latent Dirichlet allocation provides a novel interpretability of mutation signatures in cancer genomes"

Taro Matsutani and Michiaki Hamada*

1 Variational Bayes method for PLDA

Our proposed model, PLDA, is an extended model of LDA. The graphical model is shown in Figure 2 in the manuscript. Furthermore, we previously reported [1] the notation when PLDA is applied to predict mutation signatures. Those parameters in the model were learned by the variational Bayes method, and the details of the calculation are described herein.

Variational Bayes is an iterative method to update parameters to maximize the objective function, variational lower bound (VLB). Letting $q(\cdot)$ the probability of the functional argument, VLB for PLDA is calculated as follows by extending that for LDA.

$$\begin{aligned}
F[q(z,\theta,\phi \mid \boldsymbol{\xi}^{\theta},\boldsymbol{\xi}^{\phi})] &= \sum_{k=1}^{K}\sum_{v=1}^{V}\sum_{l=1}^{L}\sum_{s=1}^{S_l}\sum_{i=1}^{n_s} q(z_{lsi}=k)\delta(m_{lsi}=v)\mathbb{E}_{q(\boldsymbol{\phi}_k|\boldsymbol{\xi}_k^{\phi})}[\log\phi_{kv}] \\
&+ \sum_{l=1}^{L}\sum_{s=1}^{S_l}\sum_{k=1}^{K}\sum_{i=1}^{n_s} q(z_{lsi}=k)\mathbb{E}_{q(\boldsymbol{\theta}_{ls}|\boldsymbol{\xi}_{ls}^{\theta})}[\log\theta_{lsk}] \\
&- \sum_{l=1}^{L}\sum_{s=1}^{S_l}\sum_{i=1}^{n_s}\sum_{k=1}^{K} q(z_{lsi}=k)\log q(z_{lsi}=k) \\
&- \sum_{l=1}^{L}\sum_{s=1}^{S_l}\mathbb{KL}\left[q(\boldsymbol{\theta}_{ls}\mid\boldsymbol{\xi}^{\theta})\ ||\ p(\boldsymbol{\theta}_{ls}\mid\boldsymbol{\alpha}_l)\right] - \sum_{k=1}^{K}\mathbb{KL}\left[q(\boldsymbol{\phi}_k\mid\boldsymbol{\xi}^{\phi})\ ||\ p(\boldsymbol{\phi}_k\mid\boldsymbol{\beta})\right]
\end{aligned}$$

- $\xi_{lsk}^{\theta} = \sum_{i=1}^{n_s} q(z_{lsi}=k) + \alpha_{lk}$
- $\xi_{kv}^{\phi} = \sum_{l=1}^{L}\sum_{s=1}^{S_l}\sum_{i=1}^{n_s} q(z_{lsi}=k)\delta(m_{lsi}=v) + \beta_v$

Since $p(\boldsymbol{\theta}_{ls}\mid\boldsymbol{\alpha}_l)$ and $p(\boldsymbol{\phi}_k\mid\boldsymbol{\beta})$ are Dirichlet distributions, the fourth term of $F[q(z,\theta,\phi\mid\xi^{\theta},\xi^{\phi})]$ can be explicitly calculated. Update formulae are derived by partial differentiation of VLB for each parameter and considering the local maximum:

$$\begin{aligned}
q(z_{lsi}=k) &\propto \frac{\exp\left[\Psi(\xi_{km_{lsi}}^{\phi})\right]}{\exp\left[\Psi(\sum_{v'=1}^{V}\xi_{kv'}^{\phi})\right]}\frac{\exp\left[\Psi(\xi_{lsk}^{\theta})\right]}{\exp\left[\Psi(\sum_{k'=1}^{K}\xi_{lsk'}^{\theta})\right]} \\
q(\boldsymbol{\theta}_{ls}\mid\boldsymbol{\xi}_{ls}^{\theta}) &= \frac{\Gamma(\sum_{k=1}^{K}\xi_{lsk}^{\theta})}{\prod_{k=1}^{K}\Gamma(\xi_{lsk}^{\theta})}\prod_{k=1}^{K}\theta_{lsk}^{\xi_{lsk}^{\theta}-1} \\
q(\boldsymbol{\phi}_k\mid\boldsymbol{\xi}_k^{\phi}) &= \frac{\Gamma(\sum_{v=1}^{V}\xi_{kv}^{\phi})}{\prod_{v=1}^{V}\Gamma(\xi_{kv}^{\phi})}\prod_{v=1}^{V}\theta_{kv}^{\xi_{kv}^{\phi}-1}
\end{aligned}$$

Where $\Gamma(\cdot)$ and $\Psi(\cdot)$ show the gamma function and the digamma function, respectively.

*To whom correspondence should be addressed. Tel: +81 3 5286 3130; Fax: +81 3 5286 3130; Email: mhamada@waseda.jp

Furthermore, we used a fixed-point iteration method to update the hyperparameters. To define $\hat{\boldsymbol{\alpha}}$ as $\boldsymbol{\alpha}$ before update, the update formula for $\boldsymbol{\alpha}$ is as follows:

$$\alpha_{lk} = \frac{\sum_{s=1}^{S_l} [\Psi(\sum_{i=1}^{n_s} \delta(z_{lsi} = k') + \hat{\alpha}_{lk}) - \Psi(\hat{\alpha}_{lk})] \, \hat{\alpha}_{lk}}{\sum_{s=1}^{S_l} \left[\Psi(\sum_{k'=1}^{K} (\sum_{i=1}^{n_s} \delta(z_{lsi} = k') + \hat{\alpha}_{lk'})) - \Psi(\sum_{k'=1}^{K} \hat{\alpha}_{lk'})\right]}$$

The update of $\boldsymbol{\beta}$, which is a hyperparameter of the mutational distribution, is not different from the case of LDA.

On performing signature prediction using this method, the aforementioned parameter updates were repeated 1000 times (it was confirmed that the rise in VLB has converged in any case.). Furthermore, with the variational Bayes method, it is possible that the predicted solution becomes a local minimum; hence, we reassign the initial value of the parameter 10 times to avoid it and adopted the solution with the best VLB as the representative value.

2 Comparison with Supervised LDA

As described in Section 4.2, Supervised LDA[2] is a probabilistic model that uses auxiliary information for each sample, similar to PLDA. Figure S1 shows the graphical model of Supervised LDA. In this model, there are new variables, l_s and η. l_s shows the tumor types of sth sample. In this way, Supervised LDA regards auxiliary information as an observed random variable, and searches for parameters that fits to them in the course of learning. Additionally, η is the parameter of a probabilistic distribution generating l_s. When auxiliary information takes discrete values like this time, we think that Supervised LDA can be implemented by considering the following generation process:

$$p(l_s = x) = \frac{\xi_x}{\sum_{x' \in \Omega} \xi_{x'}}$$

$$\xi_x = \sum_{k=1}^{K} \eta_{xk} \left\{ \frac{1}{n_s} \sum_{i=1}^{n_s} q(z_{si} = k) \right\}$$

Here, x and Ω shows the tumor types and a set of them, respectively. The responsibility is weighted by η and we can get the new parameters of a multinomial distribution generating l_s. In this manner, the tumor type is treated as a random variable in the framework , and it is generated from the signature distribution (i.e. $q(z_{si})$). We believe that this modeling is not valid because causal relationship is reversed; the signature distributions are actually determined by the tumour type. PLDA parallelizes the hyperparameters (α_l) that generate the signature distribution, so this problem does not occur.

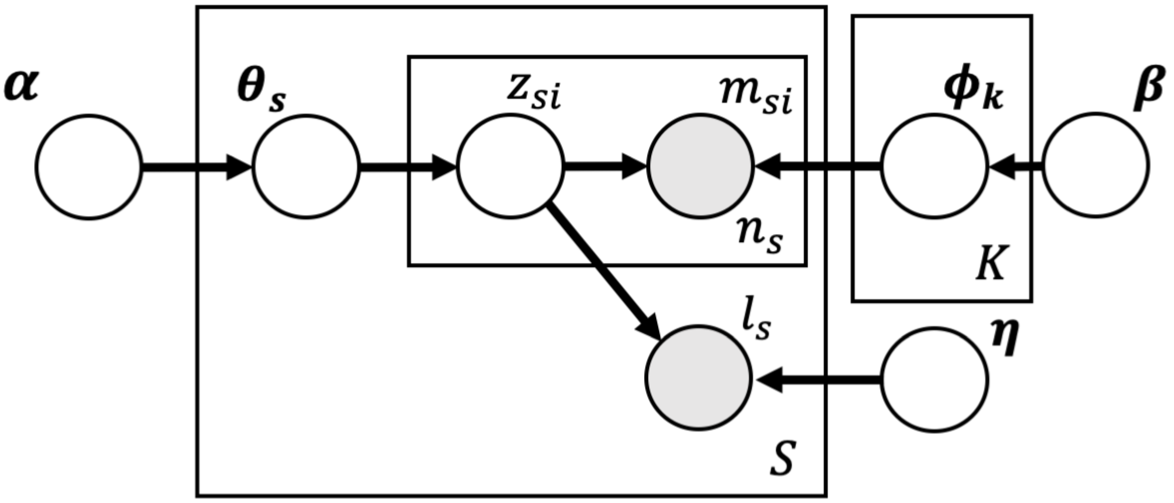

Figure S1: **The graphical model of Supervised LDA.**
This figure shows the graphical model of Supervised LDA[2]. Compared with PLDA, Supervised LDA regards auxiliary information (l_s in this figure) as an observed random variable.

3 How to create artificial mutation catalogs used in Simulation

Herein, we have described how to create an artificial mutation catalog used in the simulation experiment, as described in Section 3.1 of the paper. The purpose of this simulation experiment was to compare the performance of signature prediction between PLDA and other previous methods (normal LDA, SigProfiler, and SignatureAnalyzer). First, it should be supposed that the mutation catalog to predict signatures is a mixture of samples from $L = 5$ tumour types with quite different signature activities. As described in Table 1, the samples obtained from these tumour types are the result of SBS1 to SBS5 signatures ($K = 5$) predicted by SigProfiler [3]. In addition, each sample has $n_{ls} = 400$ mutations, and there are $S_l = 50$ samples for each tumour type (i.e. the total number of samples in one mutation catalog is $S_l \times L = 250$). The 400 mutations in each sample are generated according to the following PLDA generation process:

$$\alpha_l \in \mathbb{R}^{K=5}, 1 \leq \forall l \leq L = 5$$
$$\theta_{ls} \sim \text{Dir}(\alpha_l), \theta_{ls} \in \mathbb{R}^{K=5}, 1 \leq \forall s \leq S_l = 50$$
$$z_{lsi} \sim \text{Cat}(\theta_{ls}), z_{lsi} \in \{1, 2, \cdots, K = 5\}, 1 \leq \forall i \leq n_{ls} = 400$$
$$m_{lsi} \sim \text{Cat}(\phi_{k=z_{lsi}}), m_{lsi} \in \{1, 2, \cdots, V = 96\}$$

where each notation is the same as described in the Methods section. Only the hyperparameter α_l was artificially determined in these mutation catalogs, and we set the values so that active signatures were different for each tumor type, as shown in Table 1 (e.g., in artificial tumour type 1, SBS1 and SBS2 tended to be active, and SBS3 to SBS5 tended to be inactivated, so we set $\alpha_{l=1} = \{0.5, 0.5, 0.05, 0.05, 0.05\}$). As bias would be introduced with the use of one mutation catalog to evaluate the performance, we generated 30 mutation catalogs in the above manner and used the prediction results of signatures for all of them in the evaluation. At this time, it should be noted that even if the generation process is the same, the contents of the 30 mutation catalogs would be different because the above catalog generation is conducted by sampling with a random number seed.

4 Supplementary results of simulation with synthetic data obtained from PCAWG cohort

The Supplementary results referenced in Section 3.2 of the main text are discussed here. In addition to "1350 synthetic whole genome mutational spectra 150 spectra from each of nine cancer types" as described in Section 3.2 of the main text, we applied PLDA to the large whole-genome sequenced catalog[1] and the whole-exome sequenced catalog[2].

First, the large whole-genome sequenced mutation catalog consists of 2700 samples from nine tumor types by the contributions of 21 mutation signatures. From this synthetic mutation catalog, PLDA could predict $K = 20$ signatures, among which, 17 matched correct signatures. All the matching result are shown in Supplementary Table S1; PLDA could not extract SBS29, SBS30, SBS40, and SBS9. Supplementary Table S2 shows the comparison against the existing methods (SigProfiler and SignatureAnalyzer). Neither SigProfiler nor SignatureAnalyzer could extract SBS29 and SBS9.

Table S1: Matching result of synthetic large WGS data published from PCAWG project

Predicted	Matched	Cosine similarity	True	Matched	Cosine similarity
1	SBS28	0.9674	SBS1	3	1.0000
2	**SBS2**	0.9999	SBS13	12	0.9992
3	SBS1	1.0000	SBS15	9	0.9880
4	SBS4	0.9583	SBS17a	6	0.9999
5	SBS18	0.9929	SBS17b	17	0.9868
6	SBS17a	0.9999	SBS18	**5**	0.9929
7	SBS26	0.8526	SBS2	2	0.9999
8	**SBS5**	0.8693	SBS21	15	0.9997
9	SBS15	0.9880	SBS22	19	0.9964
10	**SBS5**	0.8790	SBS26	7	0.8526
11	**SBS3**	0.8205	SBS28	1	0.9674
12	SBS13	0.9992	<u>SBS29</u>	**5**	0.7958
13	**SBS3**	0.9311	SBS3	13	0.9311
14	**SBS2**	0.8080	<u>SBS30</u>	14	0.7666
15	SBS21	0.9997	SBS4	4	0.9583
16	SBS41	0.9854	<u>SBS40</u>	11	0.8123
17	SBS17b	0.9868	SBS41	**16**	0.9854
18	SBS8	0.8923	SBS44	20	0.9563
19	SBS22	0.9964	SBS5	10	0.8790
20	SBS44	0.9563	SBS8	18	0.8923
-	-	-	<u>SBS9</u>	**16**	0.7101

This table shows the matching results based on the cosine similarity with synthetic and large WGS data from PCAWG project, and can be interpreted in a similar manner to Table 7 in the main text. Out of 21 signatures, PLDA could extract 17 correct signatures.

Table S2: Comparison of the methods with synthetic large WGS data

Method	# Extracted (True : 21)	Avg. cosine similarity	Reconstruction rate
PLDA (proposed)	17	0.9354	0.9913
SigProfiler	19	0.9646	0.9965
SignatureAnalyzer	19	0.9582	0.9977

This table shows the comparison of the methods with synthetic large WGS data, and can be interpreted in a similar manner to Table 8.

[1] https://www.synapse.org/#!Synapse:syn18500213
[2] https://www.synapse.org/#!Synapse:syn18909829.4

Next, we applied PLDA to the whole-exome sequenced mutation catalog that consists of 2700 samples from nine tumor types by the contributions of 21 mutation signatures, whose number of mutations per sample (n_{ls}) tends to be smaller than that of WGS. When PLDA was applied to this mutation catalog, 11 identical signatures were extracted (Supplementary Table S3). Compared to the results of SigProfiler, PLDA missed 10 signatures (SBS22, SBS26, SBS28, SBS29, SBS30, SBS40, SBS41, SBS44, SBS8 and SBS9), whereas SigProfiler could not extract SBS15, SBS18 and SBS3 in addition to these 10 correct signatures (Supplementary Table S4). Additionally, SignatureAnalyzer extracted 12 signatures and successfully predicted SBS22 compared to PLDA. However, PLDA was the most accurate in terms of reconstruction rate that could be evaluated not only for the mutational distributions but also for the activities.

Table S3: Matching result of synthetic WES data published from PCAWG project

Predicted	Matched	Cosine similarity	True	Matched	Cosine similarity
1	SBS21	0.9977	SBS1	7	0.9971
2	SBS5	0.9150	SBS13	10	0.9997
3	SBS3	0.8589	SBS15	**11**	0.9212
4	SBS2	1.0000	SBS17a	5	0.9997
5	SBS17a	0.9997	SBS17b	**6**	0.9991
6	SBS17b	0.9991	SBS18	**8**	0.9808
7	SBS1	0.9971	SBS2	4	1.0000
8	SBS18	0.9808	SBS21	**1**	0.9977
9	SBS4	0.9189	SBS22	**9**	0.3977
10	SBS13	0.9997	SBS26	**1**	0.7265
11	SBS15	0.9212	SBS28	**6**	0.4462
-	-	-	SBS29	**8**	0.8254
-	-	-	SBS3	**3**	0.8589
-	-	-	SBS30	**2**	0.5129
-	-	-	SBS4	**9**	0.9189
-	-	-	SBS40	**3**	0.7539
-	-	-	SBS41	**3**	0.5920
-	-	-	SBS44	**11**	0.7731
-	-	-	SBS5	**2**	0.9150
-	-	-	SBS8	**9**	0.7510
-	-	-	SBS9	**3**	0.5279

This table shows the matching result based on the cosine similarity with synthetic WES data from PCAWG project, and can be interpreted in a similar manner to Table 7 in the main text. Out of 21 signatures, PLDA could extract 11 signatures correctly.

Table S4: Comparison of the methods with synthetic WES data

Method	# Extracted (True : 21)	Avg. cosine similarity	Reconstruction rate
PLDA (proposed)	11	0.8588	0.8149
SigProfiler	8	0.8255	0.7991
SignatureAnalyzer	12	0.8875	0.7213

This table shows the comparison of the methods with synthetic WES data, and can be interpreted in a similar manner to Table 8.

5 Model selection of PLDA

Upon parameter learning via the variational Bayes method, the number of signatures K was assumed to be known. However, we do not need know the number of signatures included in the mutation catalog, and the model can predict K automatically comparing the VLB values calculated after parameter learning when K was changed. Supplementary Figure S2 shows the results of model selection when applying PLDA to the actual mutation catalogs with whole-genome sequenced, and the model predict $K = 41$. Supplementary Figure S2 also includes the transition of the reconstruction rate, RR. Please see Section 3.2 for the definition of RR. RR takes a value from 0.0 to 1.0, and $RR = 1.0$ indicates that the original mutation catalog could be completely reconstructed. Essentially, the value of RR continues to improve by increasing the number of signatures K, because the representation power of the model increases. Therefore, we cannot use RR as an index for model selection; instead, we need to use a regularized index. From Supplementary Figure S2, we can see that the RR value is large in $K > 41$ compared with $K = 41$ (e.g. RR with $K = 41$ is approximately 0.940, but that with $K = 47$ is approximately 0.942). This indicated that the proposed method with regularization by VLB can select a model with smaller KL-divergence between the posterior distribution and the "true" distribution, rather than a model with a higher RR value (i.e. with simply a higher likelihood).

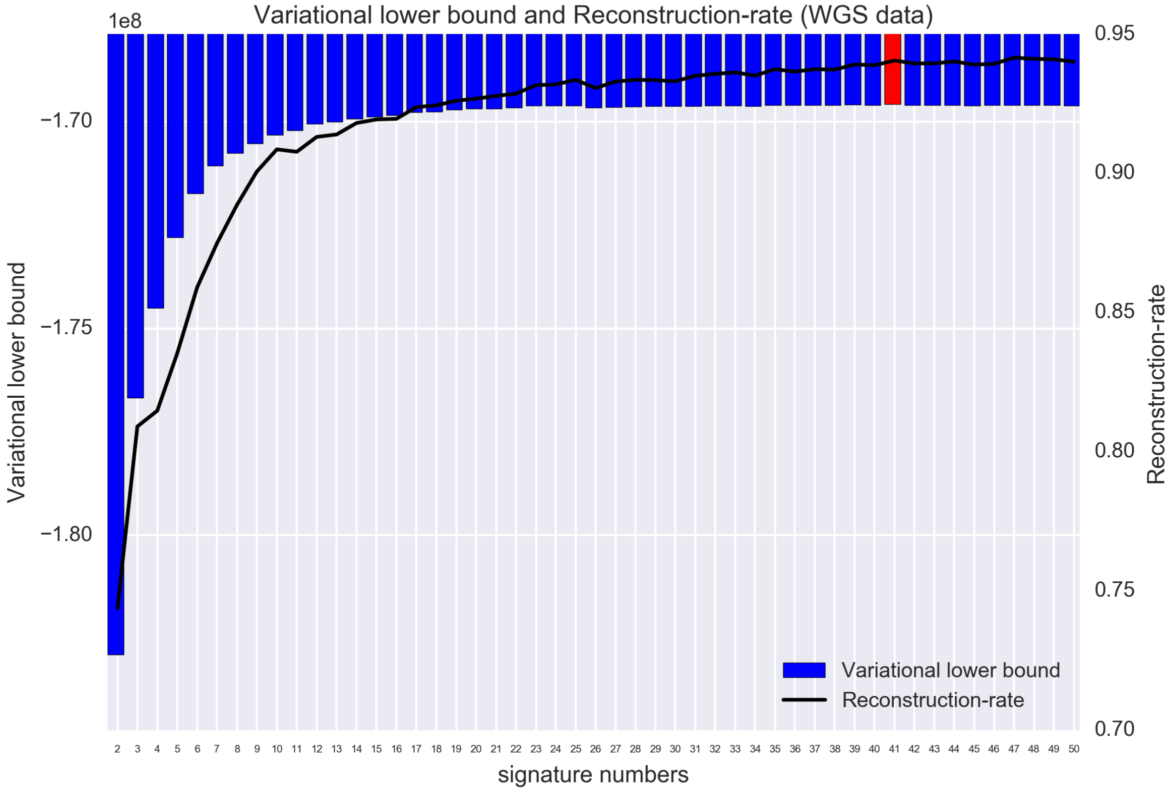

Figure S2: **Model selection of PLDA with real mutation catalogs.**
This figure shows the results of model selection when applying PLDA to the actual mutation catalogs. The horizontal axis represents the number of signatures, and the vertical axis represents the variational lower bound (VLB) value that serves as the criterion to determine the number of signatures. The bar with the largest VLB is red, and $K = 41$ is predicted. This figure also includes the reconstruction rate (black-line), and the RR value is larger in $K > 41$ compared with $K = 41$ (e.g. RR with $K = 41$ is approximately 0.940, but that with $K = 47$ is approximately 0.942). Please refer to Supplementary Section 4 how to calculate RR value in detail.

6 Other Supplementary Results with real datasets

The Supplementary Tables and Figures referenced in text are posted here.

Table S5: Matching result with real data published from PCAWG project.

Predicted	Matched	Cosine Similarity	Known	Matched	Cosine Similarity
1	SBS21	0.739	SBS1	26	0.9924
2	SBS40	0.8344	SBS2	18	0.9986
3	SBS4	0.9565	SBS3	2	0.8046
4	SBS15	0.9813	SBS4	3	0.9565
5	SBS22	0.9894	SBS5	16	0.7674
6	SBS11	0.643	SBS6	4	0.8614
7	SBS21	0.9017	SBS7a	23	0.9692
8	SBS17b	0.9417	SBS7b	41	0.9367
9	SBS10a	0.8925	SBS7c	29	0.5928
10	SBS10a	0.9242	SBS7d	1	0.6068
11	SBS13	0.9884	SBS8	3	0.7824
12	SBS12	0.796	SBS9	30	0.7428
13	SBS18	0.9127	SBS10a	14	0.9804
14	SBS10a	0.9804	SBS10b	33	0.9731
15	SBS19	0.8953	SBS11	37	0.9883
16	SBS5	0.7674	SBS12	12	0.796
17	SBS3	0.7015	SBS13	11	0.9884
18	SBS2	0.9986	SBS14	38	0.9417
19	SBS7a	0.9022	SBS15	4	0.9813
20	SBS16	0.9611	SBS16	20	0.9611
21	SBS44	0.7153	SBS17a	36	0.9654
22	SBS17b	0.6974	SBS17b	8	0.9417
23	SBS7a	0.9692	SBS18	13	0.9127
24	SBS10a	0.9639	SBS19	15	0.8953
25	SBS42	0.7216	SBS20	38	0.8005
26	SBS1	0.9924	SBS21	7	0.9017
27	SBS7a	0.9202	SBS22	5	0.9894
28	SBS37	0.7866	SBS23	37	0.8182
29	SBS7a	0.7773	SBS24	13	0.6582
30	SBS28	0.919	SBS25	5	0.7391
31	SBS26	0.876	SBS26	31	0.876
32	SBS34	0.8458	SBS27	5	0.5953
33	SBS10b	0.9731	SBS28	40	0.9741
34	SBS29	0.7454	SBS29	13	0.836
35	SBS53	0.7815	SBS30	41	0.7907
36	SBS17a	0.9654	SBS31	15	0.8716
37	SBS11	0.9883	SBS32	37	0.7689
38	SBS14	0.9417	SBS33	31	0.5342
39	SBS39	0.8815	SBS34	32	0.8458
40	SBS28	0.9741	SBS35	3	0.6426
41	SBS7b	0.9367	SBS36	13	0.8291
-	-	-	SBS37	28	0.7866
-	-	-	SBS38	35	0.7527
-	-	-	SBS39	39	0.8815
-	-	-	SBS40	2	0.8344
-	-	-	SBS41	32	0.6546
-	-	-	SBS42	25	0.7216
-	-	-	SBS43	6	0.4981
-	-	-	SBS44	21	0.7153
-	-	-	SBS45	3	0.8054
-	-	-	SBS46	36	0.7853
-	-	-	SBS47	32	0.642
-	-	-	SBS48	13	0.1789
-	-	-	SBS49	35	0.3816
-	-	-	SBS50	34	0.5948
-	-	-	SBS51	34	0.5056
-	-	-	SBS52	13	0.6385
-	-	-	SBS53	35	0.7815
-	-	-	SBS54	31	0.7695
-	-	-	SBS55	6	0.6416
-	-	-	SBS56	24	0.9161
-	-	-	SBS57	30	0.5093
-	-	-	SBS58	19	0.4555
-	-	-	SBS59	13	0.4429
-	-	-	SBS60	6	0.5348
-	-	-	SBS84	25	0.6471
-	-	-	SBS85	32	0.73

This table shows the matching result based on cosine similarity with real data from PCAWG project, and can be interpreted in a similar manner to Table 7 in the main text. Table 9 in the paper summarizes these results.

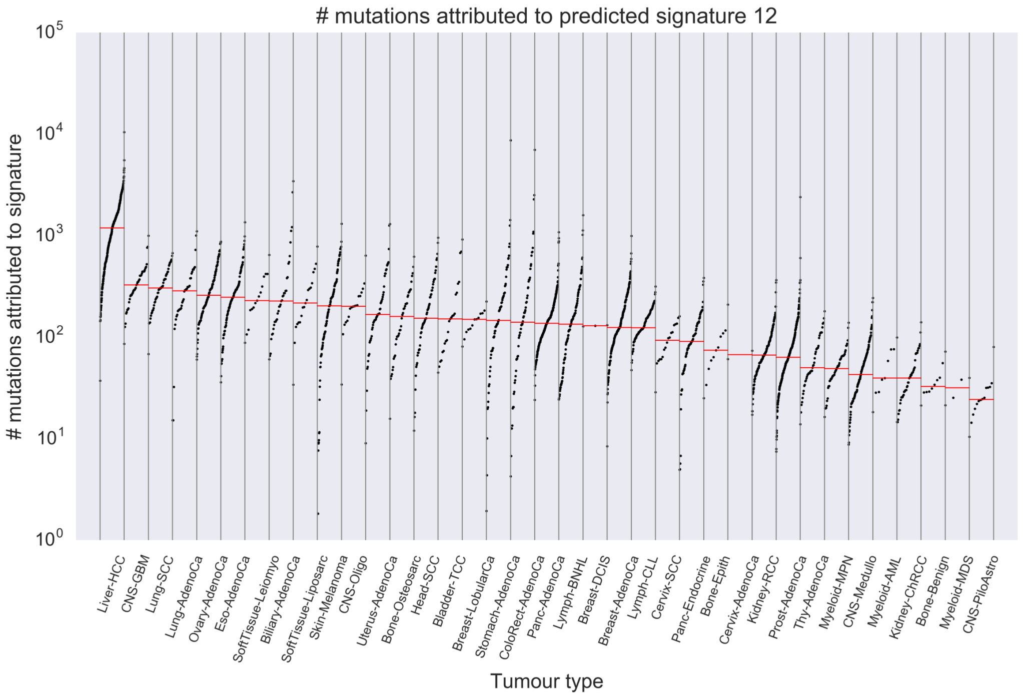

Figure S3: **The number of mutations attributed to Predicted Signature 12**
This figure shows the number of mutations attributed to **Predicted Signature 12**, and can be interpreted in a similar manner to Figure 5.

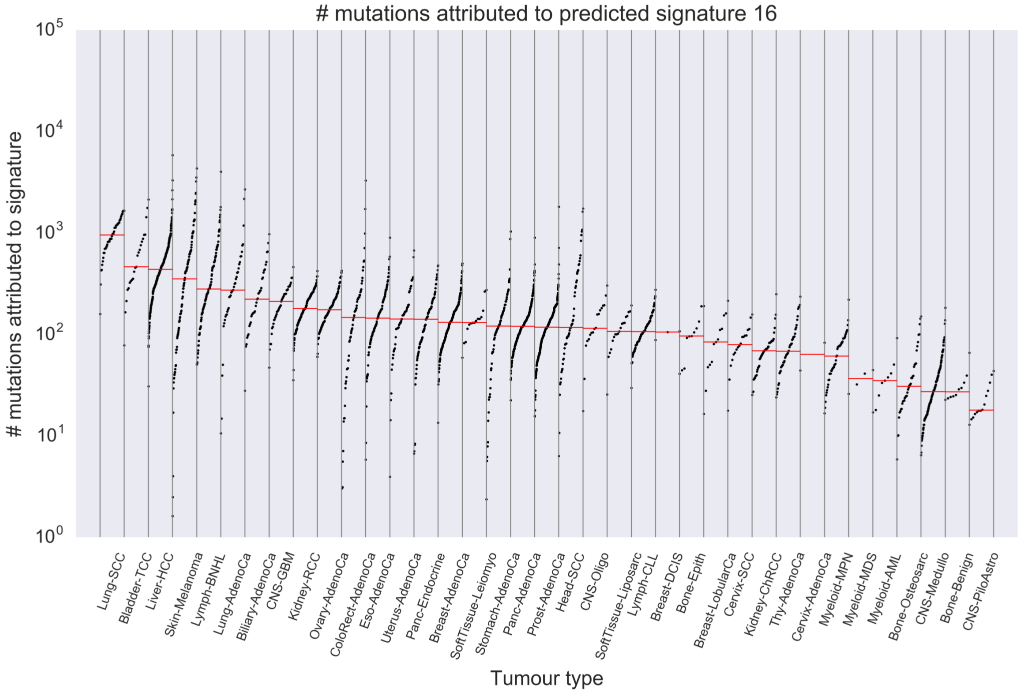

Figure S4: **The number of mutations attributed to Predicted Signature 16**
This figure shows the number of mutations attributed to **Predicted Signature 16**, and can be interpreted in a similar manner to Figure 5.

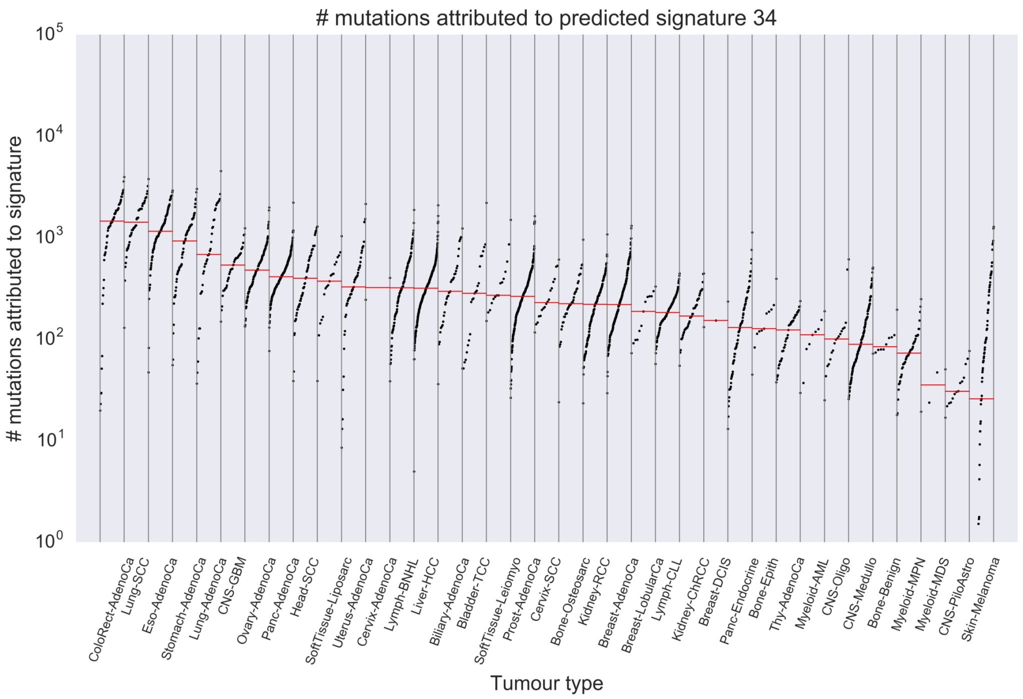

Figure S5: **The number of mutations attributed to Predicted Signature 34**
This figure shows the number of mutations attributed to **Predicted Signature 34**, and can be interpreted in a similar manner to Figure 5.

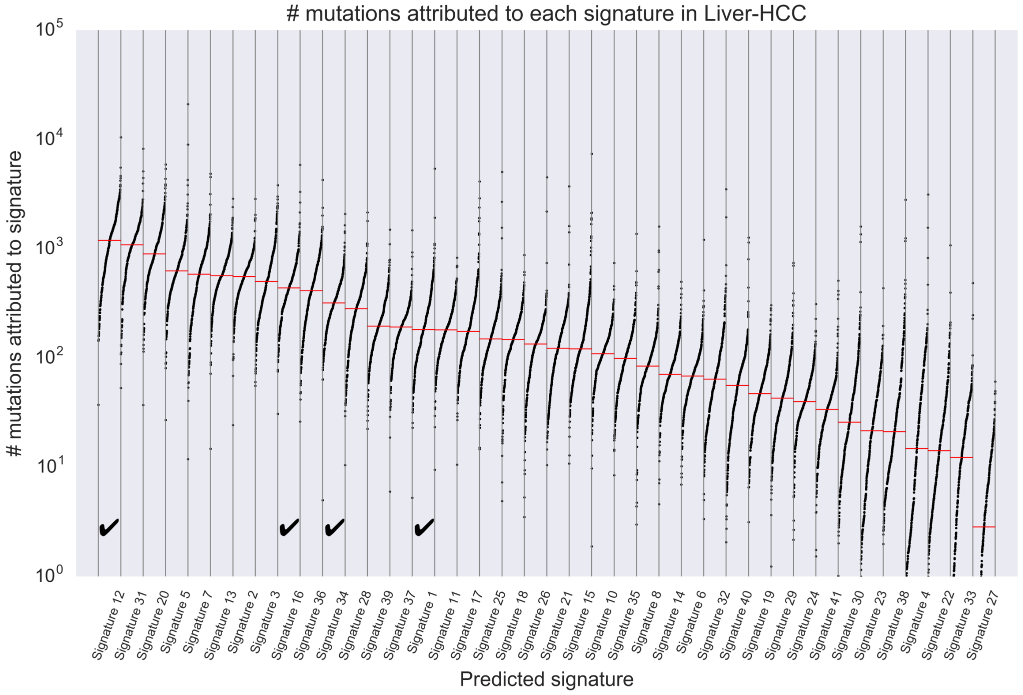

Figure S6: **The number of mutations attributed to each signature with Liver-HCC**
This figure shows the number of mutations attributed to each predicted signature in samples from **Liver-HCC**, and can be interpreted in a similar manner to Figure 6. The checked columns show the signatures of interest, which are listed in Figure 4 and have large cosine distances to most closely known signatures.

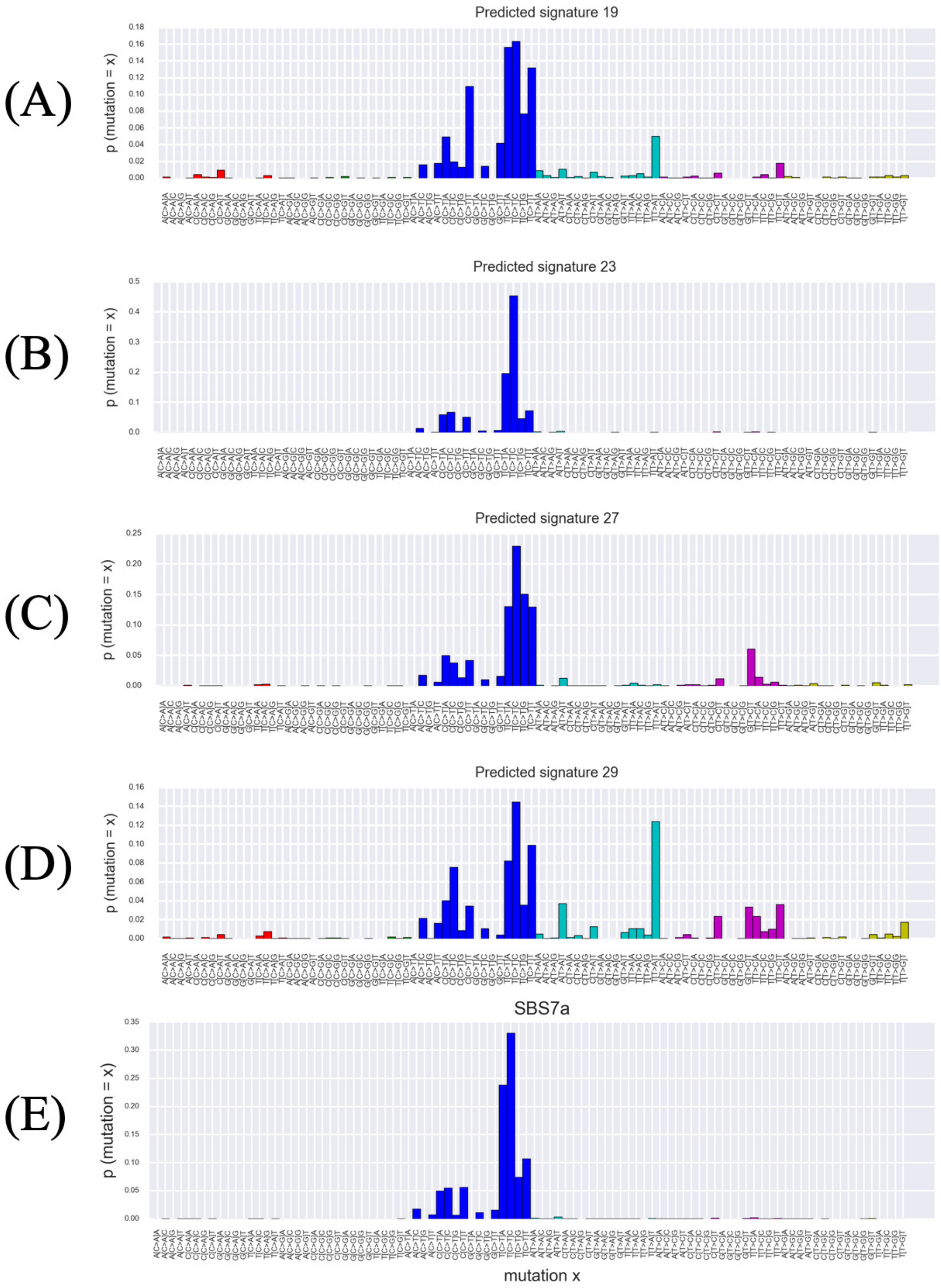

Figure S7: **Predicted Signature 19, 23, 27, 29, and SBS7a**
Each panel of (**A**)∼(**D**) shows the mutational distribution of Predicted Signatures 19, 23, 27, 29 and (**E**) shows the known signature SBS7a. Each bar graph can be interpreted similar to Figure 4. All the Predicted Signatures 19, 23, 27 and 29 are matched to SBS7a with cosine distance=$\{0.098, 0.031, 0.080, 0.223\}$, respectively. All predicted signatures have peaks at T[C>T]X and they differ in other mutations.

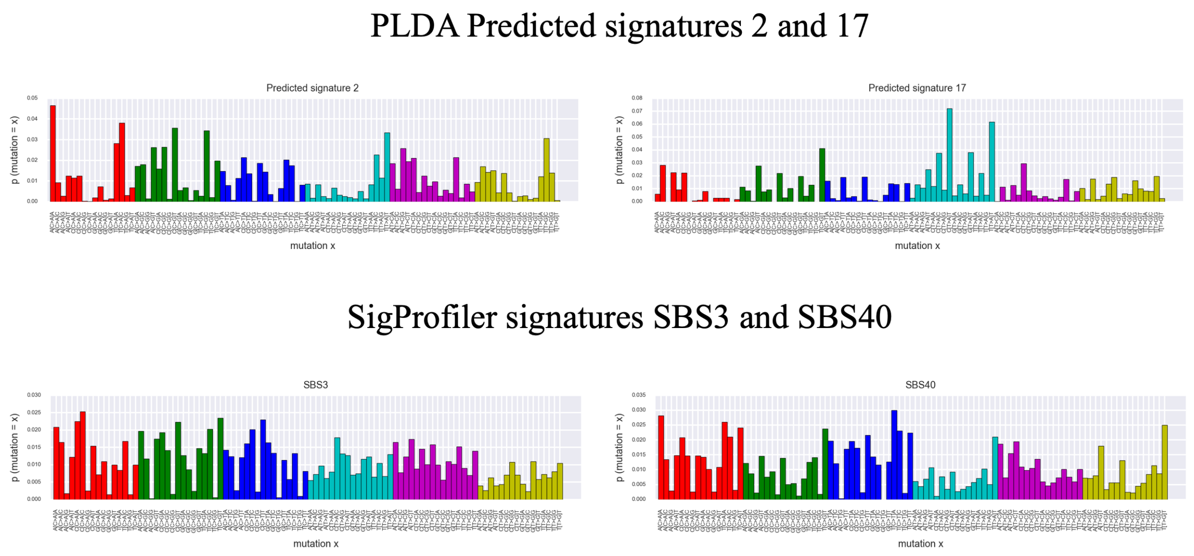

Figure S8: **Predicted Signatures 2, 17, SBS3 and SBS40**
Each panel shows the mutational distribution of Predicted Signature 2, 17, SBS3 and SBS40. Each bar graph can be interpreted similar to Figure 4. Based on the cosine distance, Predicted Signature 2 matched SBS40 (cos = 0.166), whereas Predicted Signature 17 were matched SBS3 (cos = 0.299). It can be observed that SBS3 and SBS40 had similar distributions (cosine distance 0.118); in fact, the cosine distance between Predicted Signature 2 and SBS3 was also small (0.195).

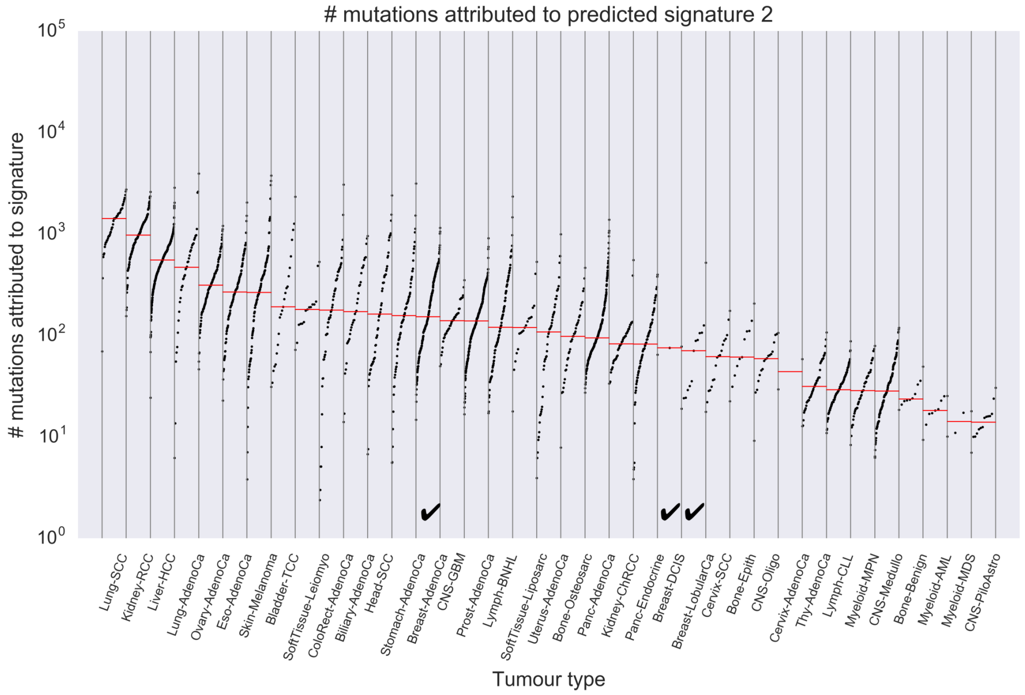

Figure S9: **The number of mutations attributed to Predicted Signature 2**
This figure shows the number of mutations attributed to **Predicted Signature 2**, and can be interpreted in a manner similar to Figure 5. The checked columns show the Breast cancer samples. SBS40 that this signature matched with, is not active in Breast cancer samples; however, SBS3 that has a similar distribution to SBS40 is active.

References

[1] Matsutani, T., Ueno, Y., Fukunaga, T., Hamada, M.: Discovering novel mutation signatures by latent dirichlet allocation with variational bayes inference. Bioinformatics (2019)

[2] Mcauliffe, J.D., Blei, D.M.: Supervised topic models. In: Advances in Neural Information Processing Systems, pp. 121–128 (2008)

[3] Alexandrov, L.B., Kim, J., Haradhvala, N.J., Huang, M.N., Ng, A.W.T., Wu, Y., Boot, A., Covington, K.R., Gordenin, D.A., Bergstrom, E.N., *et al.*: The repertoire of mutational signatures in human cancer. Nature **578**(7793), 94–101 (2020)
